# Aptamers used for molecular imaging and theranostics - recent developments

**DOI:** 10.7150/thno.72949

**Published:** 2022-05-13

**Authors:** Lennart Bohrmann, Tobias Burghardt, Charles Haynes, Katayoun Saatchi, Urs O. Häfeli

**Affiliations:** 1 Faculty of Pharmaceutical Sciences, University of British Columbia, Vancouver, BC, Canada; 2 Department of Pharmacy, Faculty of Health and Medical Sciences, University of Copenhagen, Copenhagen, Denmark; 3 Michael Smith Laboratories, University of British Columbia, Vancouver, BC, Canada

**Keywords:** *Aptamers*, * in vivo* imaging, molecular imaging, drug delivery, nanomedicine

## Abstract

Aptamers are single stranded oligonucleotides that fold into three dimensional structures and are able to recognize a variety of molecular targets. Due to the similarity to antibodies with regards to specificity and affinity and their chemical versatility, aptamers are increasingly used to create targeted probes for *in vivo* molecular imaging and therapy. Hence, aptamer-based probes have been utilized in practically all major imaging modalities such as nuclear imaging, magnetic resonance imaging, x-ray computed tomography, echography and fluorescence imaging, as well as newer modalities such as surface enhanced Raman spectroscopy. Aside from targeting, aptamers have been used for the creation of sensors that allow the localized detection of cellular markers such as ATP *in vivo*. This review focuses on *in vivo* studies of aptamer-based probes for imaging and theranostics since the comprehensive overview by Bouvier-Müller and Ducongé in 2018.

## 1. Introduction

Aptamers are short single stranded RNA or DNA-based oligonucleotides, able to bind a variety of ligands *via* non-covalent interactions, similar to antibodies (i.e., electrostatic-, hydrophobic-, Van der Waals- interactions and hydrogen bonds) 1, 2. Aptamers are typically selected against their target with an *in vitro* process called Systematic Evolution of Ligands by Exponential Enrichment (SELEX), that was first pioneered by Gold and Tuerk in 1990 3. At the same time but in independent work, Ellington and Szostak employed a similar strategy and coined the term aptamer, derived from the Greek word *aptus = to fit* 4. While the fundamental principles described by Gold and Tuerk more than 30 years ago remain in use today, more recent advancements of the SELEX methodology have allowed the selection of aptamers against a variety of targets such as small molecules, proteins, bacteria and cells within shorter timeframes 5, 6 and even the *in vivo* selection of aptamers 7-9. While the term aptamer has been used to describe both nucleic acid and peptide based combinatorial sequences 10, our review focuses on nucleic acid aptamers.

With binding affinities in the nanomolar or even picomolar range, aptamers have often been likened to antibodies for their ability to fulfil similar roles while offering a number of potential advantages including affordable and reproducible synthesis, low immunogenicity, facile chemical modification and high chemical stability 5. In contrast to antibodies, which are on the order of 150-170 kDa, aptamers are considerably smaller (12-30 kDa) and better able to diffuse through dense tissue. Xiang *et al.* were able to demonstrate better tumor penetration and retention of aptamers compared to antibodies in an *in vitro* tumor spheroid model as well as *in vivo* xenografts 11. An undeniable disadvantage that severely impedes broader *in vivo* use of aptamers, is their rapid clearance resulting in plasma half-lives in the order of minutes 12, 13. The two principal mechanisms contributing to the fast clearance of single stranded oligonucleotides are metabolic degradation by nucleases and renal excretion due to the small size of less than 5 nm for non-formulated aptamers 14.

In order to alleviate the fast clearance of aptamers, chemical modifications and the incorporation of modified nucleobases have proven to be powerful strategies to increase the circulation half-life and plasma stability of aptamers 15. Common modifications to impart nuclease resistance to aptamers include locked nucleic acids (LNAs), 2'-O-methylation, 2'-fluorination, phosphorothiolation and 3'-capping. Since 3' exonuclease activity is the predominantly responsible for deoxy oligonucleotide degradation, 3'-terminal modification of DNA aptamers can drastically improve nuclease resistance 16. RNA aptamers especially benefit from 2'-modifications such as 2'-methoxyadenine 17 or in the form of LNAs bearing a methylene bridge between the 2'-oxygen and 4'-carbon atom 18. A complete review of chemical modifications of aptamers falls beyond the scope of this review but a vast body of literature is available for consultation 15, 19, 20. Another strategy to increase the therapeutic or diagnostic value of aptamers is the conjugation to macromolecules such as polyethylene glycol (PEG) or proteins like streptavidin to increase the molecular weight and hydrodynamic diameter of the construct and thus increasing the circulation half-life. PEGylation, i.e., the conjugation of PEG chains of variable length to different classes of pharmaceuticals has long been recognized as a powerful tool to reduce clearance and prolong circulation 21. Non-surprisingly, PEGylation of aptamers is a commonly used way to prevent renal excretion and the longer circulation can promote higher uptake and retention in target tissue 22. Dougan *et al.* reported significantly improved nuclease resistance of 3'-biotinylated antithrombin aptamers *in vitro*, but no change of the *in vivo* clearance of these aptamers 23. Interestingly, aptamer-streptavidin conjugates, however, showed a 10 to 20-fold increased plasma half-life, highlighting the importance of both nuclease resistance as well as protection from renal clearance to alter the pharmacokinetic properties of aptamers. Indeed, many aptamers that underwent clinical trials are heavily modified and/or PEGylated to achieve these two goals 24-28. Pegaptanib, a modified and PEGylated RNA-based VEGF inhibitor for the treatment of age-related macular degeneration (ARMD), is arguably the most successful aptamer-based drug to date and entered the market in 2005 29. Another strategy to address the poor plasma half-life of aptamers is to employ formulation strategies like microspheres 30, nanoparticles (NPs) 31, lipid nanoparticle formulations (LNPs) 32, polymers 33 and other nanomaterials 34.

**Figure 1:**
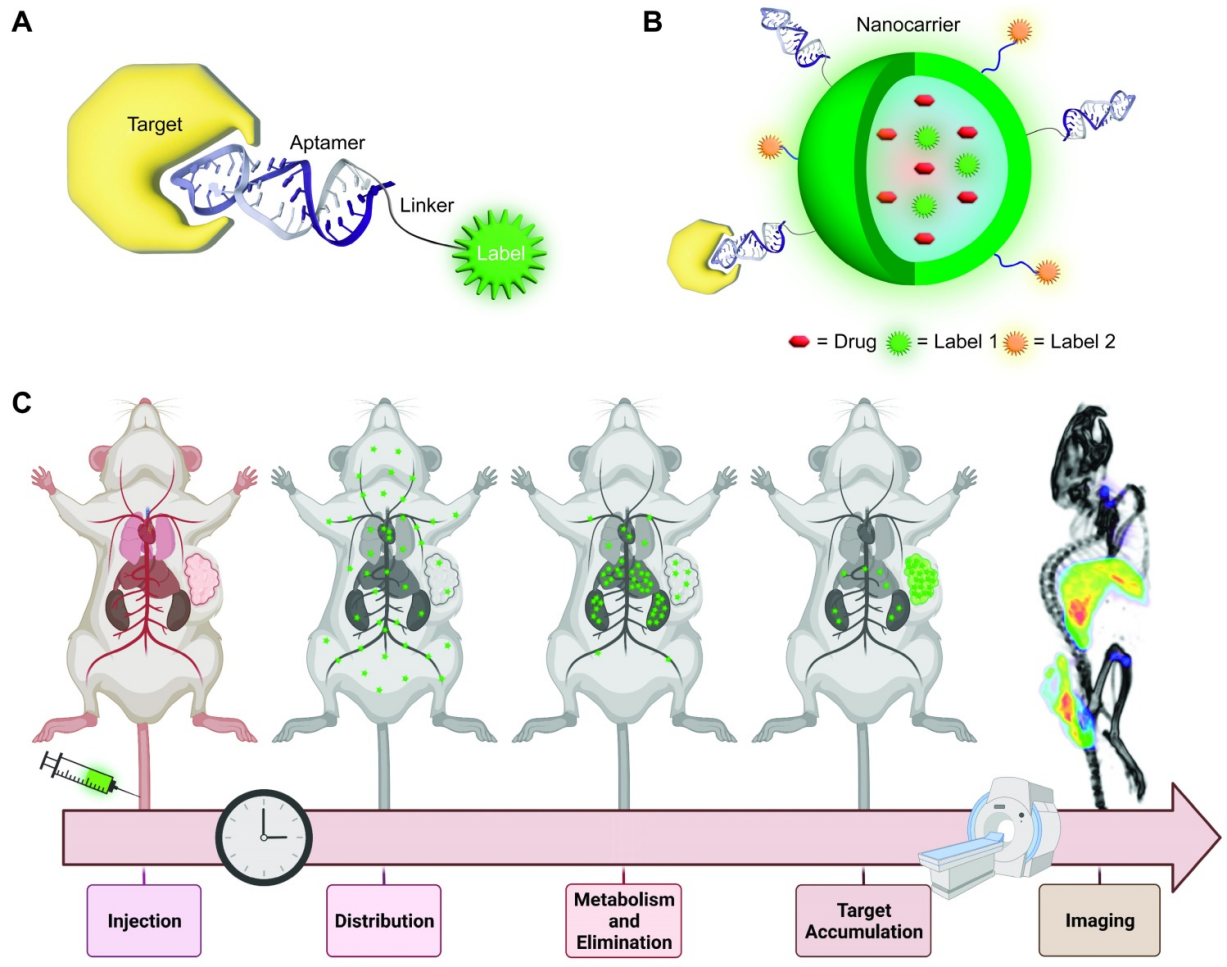
Aptamer-based probes for molecular imaging. (A) Aptamers are conjugated via a linker to a reporter molecule for molecular imaging instrument. For direct labeling fluorescent molecules or radioisotopes are especially suited. (B) Nanomaterials can serve as carrier for the aptamer, as well as one or more reporter molecules. For theranostic applications the nanocarrier can be further loaded with a drug. (C) Whole-body molecular imaging: An imaging probe is injected into the subject and distributes throughout the body. The probe accumulates at the target site (i.e., tumor) and is eliminated via the excretory organs. Imaging is performed when the target to background ratio is favorable.

Aptamers have generated considerable interest for preclinical molecular imaging with virtually all imaging modalities including Positron Emission Tomography (PET), Single-Photon Emission Computed Tomography (SPECT), Fluorescence Imaging, Magnetic Resonance Imaging (MRI), Ultrasound (US) Imaging, X-Ray computed Tomography (X-Ray CT) or combinations thereof in multimodal imaging applications 35-37. Molecular imaging probes generally consist of at least three components: i) a reporter molecule that provides a signal which can be measured by an appropriate detector, ii) a targeting moiety that specifically and selectively interacts with a molecule of interest and iii) a spacer that connects targeting ligand and reporter (Figure 1A). The spacer can furthermore be imparted with a functionality itself (e.g., redox- or pH-sensitive cleavage, ability for activation/quenching, alteration of pharmacokinetic properties of the probe and so on). With the emergence of nanomedicine, the definition of imaging probes based on these “nanomaterials” becomes increasingly difficult. In some cases, the nanomaterial itself serves as a reporter for imaging (e.g., quantum dots (QDs)), while in other cases they can serve as carrier for reporter molecules and/or therapeutic molecules or serve as a signal transducer or amplifier. The same difficulty applies for the definition of targeting moieties in this context. In some cases, the targeting moiety is covalently conjugated to the nanomaterial with or without spacers, while other formulations rely on adsorption of targeting molecules on the surface *via* electrostatic interactions (Figure 1B). Another intriguing property of nanoformulations is their ability to accumulate in tumor sites *via* the Enhanced Permeability and Retention (EPR) effect due to the increased vascular permeability in cancerous tissue 38.

The goal of molecular imaging remains the same for traditional- and nanomedicine-based imaging probes, namely gaining insight into molecular processes in a non-invasive manner. In general, this is achieved by systemic or localized administration of the imaging probe, which is followed by accumulation of the probe at the target site and excretion to achieve a high signal to background ratio (Figure 1C).

The last years saw a steady increase of imaging probes designed as theranostic agents which combine targeted therapy with diagnostics and are able to provide personalized treatment. Due to their high specificity and affinity, aptamers are ideal targeting moieties for theranostic applications and could help reduce side effects in patients by delivering therapeutic payloads precisely where they are needed and simultaneously predict treatment outcome with its diagnostic component.

Even though aptamers have been explored over 30 years as molecular imaging probes in preclinical studies, these efforts have not yet translated into clinically approved imaging probes. The situation for therapeutic aptamers is only slightly better with Pegaptanib being the only clinically approved aptamer. In this review we compile recent preclinical developments of aptamer-based molecular imaging probes (Table 1) and theranostics (Table 2) that were investigated since the comprehensive overview of the literature by Bouvier-Müller and Ducongé in 2018 39. As no human trials have been done yet, most reports are limited to preclinical models in mice unless otherwise stated. Our focus is on oligonucleotide aptamer-based probes for *in vivo* molecular imaging and theranostics that include a clear imaging emphasis. Challenges associated with the development of aptamer-based imaging probes will also be discussed, as well as the paucity of their clinical translation despite the ever-increasing number of publications in the field.

## 2. Molecular Imaging for the Characterization of Novel Aptamers

Most studies for aptamer-based imaging and theranostic probes use a disproportionally small group of established aptamers as targeting moieties 39. Worth mentioning in this regard are AS1411 40, S1.3/S2.2 41 and sgc8/sgc8c 42, 43, which target nucleolin, Mucin 1 and PTK7, respectively. Nonetheless, the discovery of novel aptamers against new targets continues at a high rate, not least due to the steadily growing toolbox of SELEX technologies. Here we focus on aptamers that have been developed for imaging applications and were evaluated *in vivo*. First tests to confirm binding and specificity in a living organism are often done with fluorescently labeled aptamers, which represent early stages of preclinical research and will require extensive modifications and formulation strategies to overcome the rapid clearance of aptamers. The use of standard fluorophores is not readily transferable to larger organisms, let alone humans for whole body imaging. Nevertheless, their use adds valuable data beyond pure *in vitro* studies and might help to identify potential aptamers against novel relevant molecular targets. In the following paragraphs recently reported novel aptamers that have been fluorescently labeled for binding validation *in vivo* are discussed 44-53. Due to the nature of cell-SELEX, the molecular target of aptamers is often not precisely known. Hence, the selected aptamers might bind other cell lines that share a common molecular target as well. For this reason, evaluating aptamers against other cell lines can be beneficial and is discussed in two examples as well 54, 55. Novel aptamers for other imaging modalities or theranostics are described in more detail in their respective sections and will not be discussed here 9, 55-60.

Hepatocellular carcinoma (HCC) is a prevalent form of cancer and one of the leading causes of cancer related deaths 61. Aptamers have previously been explored for detection, drug delivery and therapy of HCC, but the majority of studies have been limited to *in vitro* tests and no imaging has been performed to date 62. A novel addition to the list of HCC specific aptamers comes from the research groups of Weizhong Wu and Jia Ling who published several papers about a Glypican-3 (GPC3) binding DNA aptamer termed AP613-1 and used it for fluorescence imaging and MRI (see also section 3.1) 44, 45, 56. AP613-1 was selected by capillary electrophoresis against recombinant human GPC3 with a K_d_ of 59.85 ± 15.39 nM. Flow cytometry and confocal microscopy demonstrated that AP613-1 specifically binds to GPC3 expressing Huh-7 cells but not to GPC3 negative A549 cells. Unilateral Huh7 bearing mice injected with AP613-1 had 1.6-fold higher fluorescence signal in the tumor than control animals that were injected with Alexa Fluor 750-labeled control ssDNA. Specificity was confirmed in bilateral Huh7 and A549 xenograft bearing mice, showing 1.7-fold higher uptake in GPC3 positive tumors 44. In a follow up study six nucleobases in the sequence of AP613-1 were replaced with locked nucleic acids or phosphorothioate backbones to generate APS613-1 with an improved K_d_ of 15.48 ± 2.96 nM 45. A cause of concern however, is the non-negligible affinity of APS613-1 to GPC3 negative cell lines L02 and A549 with a K_d_ of 134.8 ± 49.7 and 128.1 ± 41.3 nM, respectively, raising some concern regarding off-target binding 63. This was further corroborated by some unspecific staining of L02 and A592 cell membranes. Nevertheless, the inclusion of binding dissociation constants against off-target cell lines is a welcome exception to the majority of the literature, where such information is usually not reported. Interestingly, the plasma stability of phosphorothiolated APS613-1 over 4 h is comparable to the parent sequence, warranting further research over longer periods. Fluorescence imaging of Alexa Fluor 750-labeled APS613-1 and control ssDNA in Huh7 xenografts showed high background fluorescence for the initial 120 mins; only after 150 min was tumor uptake 2.6-fold higher than the controls. In animals bearing bilateral Huh7 and A549 xenografts, a 3.3-fold higher uptake in Huh7 tumors was observed after 90 min. In a follow up study AP613-1 was conjugated to magnetic nanoparticles as MRI contrast agents, which is discussed in section 3.1 56.

A series of breast cancer specific DNA aptamers against HER2-enriched SK-BR-3 (sk6Ea) and luminal A subtype MCF-7 cells (MF3/MF3Ec) were selected by Nongyue He *et al.* 46-48. Molecular subtype differentiation is a key aspect of personalized medicine in breast cancer and the availability of subtype-specific aptamers could potentially enable the development of novel diagnostic and therapeutic probes. The selection of the aptamers followed cell-SELEX general strategy against the target cell line followed by counterselection against non-target breast cancer cell lines and normal tissue. The SK-BR-3 cell specific aptamer sk6Ea with a K_d_ 49.32 ± 14.53 nM was able to distinguish patient derived HER2-enriched breast cancer tissue from triple-negative, luminal A and B subtype and normal tissue. However, some unspecific binding of sk6Ea and ssDNA control was noticeable in all tested tissue sections, highlighting difficulties that still impede widespread use of aptamers in clinical applications such as immunohistochemistry. Fluorescence imaging of tumor mice with Cy5-labeled sk6Ea and control ssDNA in SK-BR-3, MCF-7 and MDA-MB-231 xenografts showed specific uptake for up to 5 h post injection (p.i.) only in SK-BR-3 46.

*In vivo* imaging of the MCF-7 specific aptamer with a K_d_ of 82.25 ± 25.14 nM was performed analogous to sk6Ea and revealed moderate tumor uptake in MCF-7 xenografts but no uptake in SK-BR-3 or MDA-MB-231 tumors 47. In a follow up study, MF3 was truncated to generate MF3Ec with a 4-fold lower K_d_ of 18.95 ± 2.9 nM against MCF-7 cells 48. Imaging with MF3Ec was possible up to 5 h and the aptamer was stable in plasma for up to 12 h. Molecular subtyping of breast cancer tissue with MF3Ec positively identified luminal A subtype from other breast cancer types with similar non-specific binding as sk6Ea. These studies with exemplary controls and thorough *in vitro* characterization are a promising first milestone towards the development of aptamers for molecular subtyping and targeting of breast cancer. Further research is now needed to develop imaging probes based on these aptamers.

A potential broad cancer specific aptamer, termed PS-ZL-7c, was selected by cell-SELEX against multidrug resistant HepG2/MDR cells 49. Flow cytometry showed binding against other drug resistant cancer cell lines but no binding to their respective parent cell lines. Due to phosphorothiolation, the aptamer demonstrated an impressive plasma half-life of 113 h *in vitro* and was able to specifically accumulate in HepG2/MDR tumors but not in HepG2 tumors of bilateral tumor xenografts 5 h post injection.

A novel lung-metastatic osteosarcoma specific aptamer termed LP-16 with a K_d_ of 56.73 ± 7.75 nM against 143B cells was used for fluorescence imaging of osteosarcoma xenografts and immunohistochemistry of patient derived tissue samples. I*n vitro* characterization and immunohistochemistry suggest selective binding which was confirmed by moderate but stable tumor uptake over 2 h in 143B xenografts 50.

In an effort to develop a probe that specifically targets vemurafenib-resistant melanoma, Jing Liu *et al.* selected the DNA aptamer LL4A against vemurafenib-resistant Mel28-PLX cells followed by sequence optimization through truncation 51. CD63 was identified as the molecular target of LL4A and the aptamer binds to recombinant CD63 with a K_d_ of 84.63 ± 13.04 nM. *In vivo* and *ex vivo* fluorescence imaging with Cy5-labeled LL4A was performed in mice bearing bilateral Mel28 and Mel28-PLX (vemurafenib-sensitive and resistant, respectively). Fluorescence in the Mel28-PLX tumor was detected as early as 5 min p.i. and remained detectable until 120 min. Despite the preliminary nature of the study, these findings indicate the possibility to use LL4A as a theranostic probe to monitor vemurafenib resistance or as targeting moiety for therapeutic or diagnostic applications.

A papillary thyroid carcinoma (PTC) specific aptamer termed TC-6 using was selected *via* tissue SELEX of PTC tissue samples 52. Despite being able to bind PTC cell lines and tumor sections, moderate binding to ductal breast cancer tumors as well as cervix, breast and lung cancer cell lines requires further elucidation of the specificity profile. Nevertheless, TC-6 showed moderate tumor uptake in TPC-1 xenografts after 60 min and relatively constant levels from 120 to 240 min, while no tumor uptake was observable with control DNA. The pharmacokinetic profile of TC-6 and the library matches the expected result for a non-formulated aptamer, exhibiting predominantly renal excretion.

A specifically designed library to promote duplex stabilized G-quadruplex formation was used for the selection of the aptamer BG2 53. Interestingly, although HeLa cells were used for selection, stronger binding was observed against MCF-7, LoVo, HepG2 and SMC-7721 cells. Stable isotope labeling with amino acids in cell culture (SILAC) 64, 65 identified alkaline phosphatase heterodimers as the molecular target of BG2. While the specific function of homo- and heterodimers of alkaline phosphatase is not precisely known, it is well established that dimerization plays a crucial role in protein regulation and non-native oligomerization can have pathophysiological consequences 66. This serendipitous finding demonstrates the ability of aptamers to recognize protein heterodimers due to their three-dimensional structure, highlighting the diverse nature of molecular targets for aptamers. The ability to bind alkaline phosphatase heterodimers was confirmed by *in vivo* and *ex vivo* fluorescent imaging of mice bearing LoVo xenografts. Alexa Fluor 647 labeled BG2 showed excellent tumor uptake both *in vivo* and *ex vivo* and no uptake in alkaline phosphatase negative PC3 xenografts.

The RNA aptamer E3 was originally selected against prostate cancer and showed promising results as aptamer-drug conjugate to auristatin for this condition 67. Gray *et al*. further characterized E3 as a broad cancer targeting aptamer against a large variety of other cancer cell lines, including breast, lung, pancreatic and skin cancer lines, as well as patient derived colorectal cancer, cholangiocarcinoma, renal cancer and osteosarcoma 54. While the majority of experiments were performed *in vitro* using flow cytometry, a patient derived CRC119x colorectal tumor xenograft model was established to confirm the targeting ability on E3 *in vivo*. Strong tumor uptake with low background fluorescence was observed after 48 h, with no tumor uptake for a control sequence.

R13 is a DNA aptamer that was originally selected against EGFR-GFP overexpressing A549 lung cancer cells with a K_d_ of 23 nM 68. Originally developed in 2013, R13 has been used to create fullerene nanoparticles for tumor targeted photodynamic therapy (PDT) 69 and to capture circulating tumor cells 70. Lanquin Cao *et al*. assessed the binding of R13 against a panel of ovarian cancer cell lines and found binding affinities of 47.48 ± 7.15, 29.24 ± 8.55, 37.87 ±4.93 and 158 ± 28.22 nM against HO8910, A2780, SKOV3 and Caov3, respectively 55. Furthermore, fluorescently labeled R13 stained the majority of stage I-IV ovarian cancers but not normal tissues in a patient derived microarray. Although the molecular target of R13 is still unidentified, the aptamer seems to bind extracellular targets and undergoes endocytosis *via* clathrin- and caveolae-mediated uptake. Its targeting ability was further examined in A2780 xenografts. Tumor uptake was observable after 1.5 h and a weak signal remained even after 11.5 h.

Similar to the just described R13 or E3 aptamers, other aptamers that were generated by cell-SELEX might be repurposed for other indications. This highlights yet again the importance of identifying and characterizing the molecular targets of existing aptamers, which is unfortunately often neglected.

## 3. Modalities for aptamer imaging

### 3.1 MRI

Magnetic resonance imaging is a non-invasive imaging technology with high spatial resolution in the order of 10^-5^ m that produces three-dimensional anatomical images based on the concentration of protons in a given tissue 71, 72. The contrast obtained from the differences in relaxation of various tissues can be increased through the introduction of exogeneous contrast agents. These contrast agents generally shorten the longitudinal (T_1_) and/or transverse (T_2_) spin relaxation time of hydrogen protons of bulk water in an external magnetic field after application of a radiofrequency pulse. According to the ratio to which a contrast agent affects the longitudinal and transverse relaxation the contrast agents are categorized in T_1_ agents, that lead to an increase in signal and bright images, or T_2_ agents, that cause darkening of the image. MRI contrast agents can furthermore be grouped by their composition or magnetic properties. Paramagnetic compounds are generally T_1_ weighted imaging agents and include chelates and nanoparticles of Gd^3+^, Mn^2+^ and Fe^3+^. In contrast superparamagnetic and ferromagnetic compounds are mostly T2 contrast agents. They are categorized based on their size into ultra-small superparamagnetic iron oxide nanoparticles (USPIONs), superparamagnetic iron oxide nanoparticles (SPIONs) and micron-sized particles of iron oxide (MPIO) 72-75. Currently chelates of gadolinium and manganese, as well as SPIONs are clinically approved. However, all these contrast agents rely on passive distribution and accumulation, which is a reason for extensive research on targeted contrast agents 76. In the following paragraph, recent developments of aptamer-targeted MRI contrast agents are discussed.

**Figure 2:**
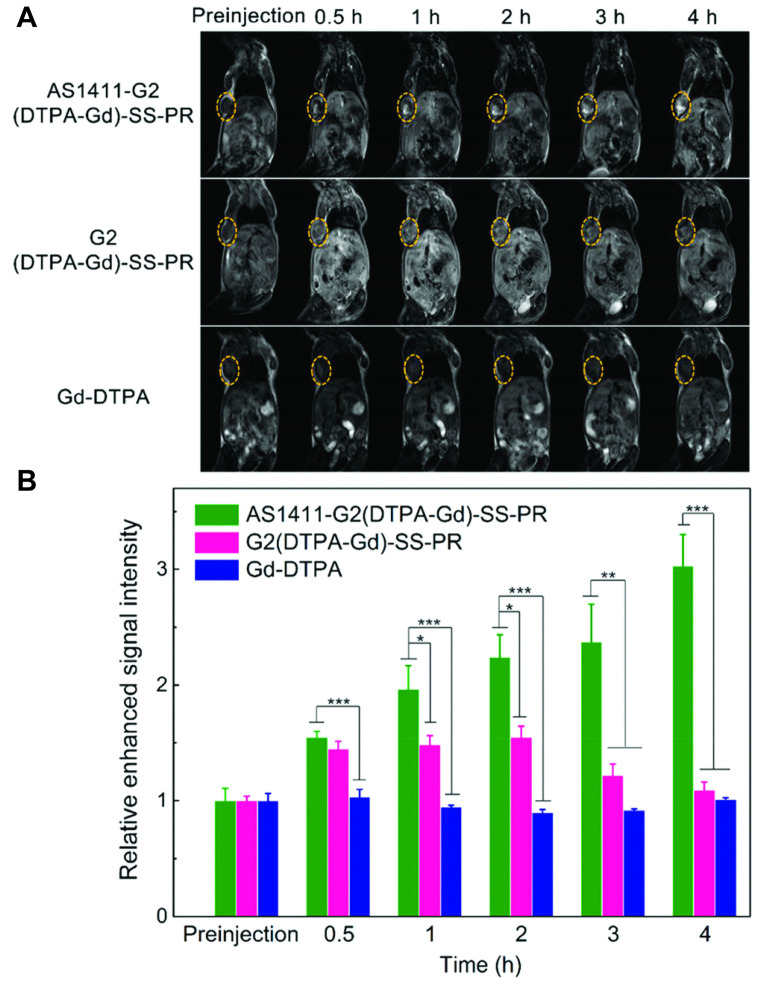
MRI and image analysis of mice injected with polyrotaxane contrast agents. (A) T_1_-weighted images of MCF-7 tumor bearing mice injected with AS1411-targeted nanoprobe (top), untargeted nanoprobe (middle) and Gd-DTPA (bottom) over time. (B) Relative enhanced signal intensity of tumor region over time normalized to preinjection intensity. Adapted with permission from 77. Copyright 2019 American Chemical Society.

Zu *et al.* reported an AS1411-targeted polyrotaxane-based contrast agent for MRI 77. The probe consists of multiple Gd-DTPA-labeled α-cyclodextrin subunits threaded on a PEG backbone which is end-capped with benzyloxycarbonyl-L-tyrosine moieties *via* a reducible S-S bond. The limited molecular rotation in combination with the high gadolinium content of the probe increases the relaxivity (11.7 mM^-1^ s^-1^) of the contrast agent compared to free Gd-DTPA (4.16 mM^-1^ s^-1^). The contrast enhancement of the AS1411-targeted and non-targeted polyrotaxane, as well as Gd-DTPA was assessed in MCF-7 xenograft bearing mice after intravenous injection of 0.1 mmol/kg Gd^3+^ per group. AS1411-targeted polyrotaxane increased the contrast 3-fold after 4 h, while untargeted polyrotaxane resulted in a transient and moderate signal enhancement that peaked after 2 h and returned to baseline by 4 h. No signal enhancement was observed for the group injected with Gd-DTPA (Figure 2). This result suggests aptamer dependent tumor accumulation and not just passive accumulation *via* the EPR effect. Due to the disulfide bond, the end capping groups are cleaved under reducing conditions and the cyclodextrin subunits can dissociate from the PEG-backbone, resulting in accelerated clearance. Consequently, after 10 days the Gd^3+^ retention in major organs was comparable to other biodegradable polymeric contrast agents 78, albeit slightly higher than small molecule Gd-chelates like Gd(DTPA), Gd(DOTA) or Gd(acetate) 79, with the highest concentration found in the kidneys.

Two novel DNA aptamers against the neovascularization biomarker endoglin, termed Apt1 and Apt2 with a K_d_ of 98 pM and 132 pM, respectively, were identified using conventional SELEX 55. Apt1 was conjugated to fluorescently labeled and Gd-DTPA functionalized fifth-generation PAMAM dendrimers to afford a multimodal nanoprobe for MRI and fluorescence imaging. The nanoprobe showed almost two-fold higher uptake by endoglin overexpressing SMMC-7721 cells compare to untargeted dendrimer and uptake could be blocked in competition experiments with excess endoglin antibody. For *in vivo* MRI imaging, SMMC-7721-GFP tumors were grown subcutaneously in mice and implanted onto the liver of another group of nude mice. This complex but more meaningful orthotopic tumor model presents a notable exception from the majority of preclinical studies for molecular imaging probes, where subcutaneous tumor models are used. Apt1-targeted nanoprobes, non-targeted nanoprobes and Apt1-targeted nanoprobes after pre-treatment with endoglin antibody were intravenously injected at a concentration of 0.05 mmol/kg Gd^3+^. The signal to noise (S/N) ratio of the targeted and non-targeted nanoprobe reached 2.11 and 1.68 after 24 h, respectively, whereas pre-treatment reduced the S/N ratio to 1.24. Similar results were found by *ex vivo* NIR-fluorescent imaging. Immunofluorescence imaging of resected tumors further showed co-localization of the Apt1 nanoprobe and endoglin.

Kai Xu *et al.* published a series of papers about design and synthesis strategies of AS1411 targeted nanoprobes for MR imaging of renal cancer. Interestingly, these probes allow for multimodal imaging due to the use of quantum dots (QDs) with fluorescent properties, although only MRI was assessed *in vivo*. Aptamer-based probes for *in vivo* fluorescent imaging are further discussed in section 4.In a proof-of-concept study they explored the intriguing possibility of creating T_1_ and T_2_ dual imaging agents 80. This was realized by using mesoporous silica as carrier for bovine serum albumin (BSA)-Gd_2_O_3_ and Fe_3_O_4_ NPs. The high T_1_ and T_2_ relaxivity of 11.5 s^-1^ mM^-1^ and 195.1 s^-1^ mM^-1^, respectively, was achieved through a 20 nm mesoporous silica shell that spatially separated the contrast agents and thereby reduced interference. Regrettably, AS1411 targeted NPs were only used for *in vitro* experiments to demonstrate that the construct can be specifically targeted to 768-O renal cancer cells. MRI was only performed in healthy mice injected with untargeted NPs. Images showed T_1_ and T_2_ contrast enhancement in the kidneys within 15 minutes and prolonged signal in the bladder, suggesting a predominantly renal excretion pathway despite the relatively large size of 345.6 nm of the probe. The results demonstrate that combined T_1_ and T_2_ contrast enhancing agents with good biocompatibility are potentially feasible, although specific targeting should be evaluated* in vivo*.

In another approach, 1.9 nm sized MnO NPs coated with carboxy functionalized PEG were conjugated to 5'-amine modified AS1411 *via* EDC/NHS chemistry 81. The nanoprobe serves as a contrast agent in T_1_ weighted MRI and displayed good relaxivity with an r_1_ value of 12.942 s^-1^ mM^-1^. Important characteristics for nanodiagnostics such as *in vitro* and *in vivo* toxicity, biostability and circulation half-life, however, were evaluated only for the untargeted probe. *In vivo*, the aptamer-targeted and non-targeted NPs displayed very similar pharmacokinetics and signal intensity in the examined organs (liver, kidney, muscle, and tumor) in subcutaneous 786-O xenograft bearing mice, suggesting negligible impact of AS1411 for tumor accumulation and retention. The highest tumor signal intensity was achieved within the first hour of administration, followed by a decrease in signal intensity, which falls in line with the plasma circulation of 60 min for the nanoprobe.

AS1411-modified Mn-doped MoS_2_ QDs were used for multimodal fluorescence and MRI of renal cell carcinoma 82. With a T_1_ relaxivity of 16.95 mM^-1^ s^-1^ that exceeds the relaxivity of Gd-DTPA by a factor of four and excitation and emission maxima of 290 and 380 nm, respectively, the quantum dots exhibited excellent properties for MRI and *in vitro* fluorescent imaging. The QDs had selective toxicity against a range of cancer cells (786-O, MDA-MB-231 and HO-8910), while no cytotoxicity was observed for normal cells (EA.hy926 and HK-2). This intriguing and noticeable anticancer effect was attributed to altered pH and hydrogen peroxide levels in tumor cells that lead to Mn catalyzed production of oxygen, but further evaluation of this preferential toxicity towards cancer cells is needed. T_1_ weighted MRI images of mice bearing subcutaneous 768-O xenografts showed contrast enhancement in the tumor region peaking at 1.5 and 2 h post injection of AS1411- or non-targeted QDs, respectively, with consistently higher signal intensity in the targeted group. As expected for NP formulations, high liver and notably gallbladder uptake was observed, indicating hepatobiliary excretion of this formulation.

Yet another Mn-based contrast agent with the intriguing capability of glutathione- (GSH) dependent contrast enhancement consists of MoS_2_ QDs supported on MnO_2_ nanosheets 83. The AS1411-targeted multimodal probe works through GSH-mediated reduction of the MnO_2_ nanosheet, which releases Mn^2+^ and reduces quenching of the QDs.* In vitro* experiments on 786-O cells treated either with the GSH synthesis enhancer α-lipoic acid or the GSH-scavenger N-methylmaleimide confirmed the GSH-dependent increase of the MR signal. MRI of AS1411 targeted nanosheets showed significantly higher T_1_ contrast in the tumor region in comparison to non-targeted nanosheets up to 6 h. High background signal in liver and gastrointestinal system persisted throughout the imaging period and ICP-MS confirms strong hepatobiliary retention of molybdenum in the liver, once again suggesting hepatobiliary excretion of the probe.

Aptamer targeted SPIONs were reported in a well-designed and characterized study by Heo *et al.* for MRI of Her2 positive tumors 84. The Fe_3_O_4_ nanocrystals were coated with Tween 80 and APT_HER2_ aptamer *via* maleimide-thiol coupling. The aptamer has a half-life of 151 h in human serum, due to the incorporation of 5-naphthylmethylaminocarbonyl-modified deoxyuridine nucleotides. The dissociation constant of 0.57 ± 0.26 nM for the resulting NPs barely deviated from the K_d_ of unmodified aptamer, which was measured as 0.42 ± 0.05 nM against Her2 protein in a radioligand assay. The targeted NPs showed 10-30% darkening of tumor areas in T_2_ weighted MRI imaging after injection compared to pre-injection levels in syngeneic NIH3T6.7 tumor animals. Contrary to previous studies in rat glioma 85, no signal enhancement of non-targeted NPs due to passive EPR mediated accumulation was observed. This is a notable exception to the majority of the here presented studies of NP-based imaging agents, where differences between aptamer-targeted and non-targeted nanoprobes are often less pronounced. These encouraging results could pave the way towards novel potent T_2_ contrast enhancing imaging agents using aptamer targeted SPIONs.

The previously mentioned aptamer AP613-1 was used for targeting of USPIONs as a T_2_ MRI probe for hepatocellular carcinoma 56. MRI of Huh-7 xenograft bearing mice revealed a roughly 40% relative signal enhancement within 1 h of injection, that stayed relatively constant up to 4 h, whereas no change in signal intensity was observed in control animals injected with non-targeted USPIONs. Although the *in vivo* MRI results of the aptamer targeted USPIOs look promising, it is difficult to draw a conclusion due to the lack of stringent controls. The NPs were only tested in Huh7 xenografts and a control group consisting of a scrambled aptamer sequence would be more meaningful than untargeted USPIOs. It is, however, interesting that similar to the findings of Heo *et al.* 84, no EPR mediated uptake of USPIOs seems to take place.

Zhong *et al*. reported the selection of the EpCAM specific DNA aptamer Eppc6 and conjugated it to GoldMag NPs for MRI imaging 86. The aptamers were selected using cell-SELEX against EpCAM expressing HEK293T cells, with normal HEK293T for counterselection. Binding affinity was confirmed using flow cytometry against EpCAM expressing prostate cancer cell lines PC-3, DU145 and LNCaP. MRI was performed mice bearing subcutaneous PC-3 xenografts. Compared to mice that were injected with scrambled ssDNA conjugated GoldMag, T_2_ weighted images showed a roughly 2-fold increased contrast of Eppc6-conjugated GoldMag NPs 1 h post injection, which further increased by a small but statistically significant amount at 6 h and remained at this level up to 12 h. Unfortunately, no size or other characterization of the GoldMag probes were provided to explain the obvious absence of EPR mediated tumor accumulation of the scrambled control group.

### 3.2 Nuclear Imaging

Nuclear imaging employs probes that are radioactively labeled. Depending on the type of radioactive decay, different modalities are used to detect the radiation from such “radiotracers”. In Single Photon Emission Computed Tomography (SPECT), multiple 2D projections from different angles are recorded and reconstructed to generate a 3D image containing spatial information about the gamma-emitting radiotracer(s) in an organism. Positron Emission Tomography (PET) uses positron-emitting radionuclides which, upon annihilation with an electron, produce two 511 keV photons at an angle of approximately 180°. These coincident photons are detected with gamma detectors that circularly encompass the study object and allow a 3D reconstruction of the radiotracer. Due to the higher number of photons that reach the detector in PET, the sensitivity is generally better than in SPECT. Radioisotopes required for PET imaging, however, are usually short lived and often need to be produced with expensive cyclotrons which requires substantial financial investments and logistics. The high sensitivity of nuclear imaging, allows the use of much smaller amounts of imaging agent than for MRI, CT or US 87. Consequently it is not necessarily required to attach the aptamer to a nanocarrier and it might be sufficient to attach a bifunctional chelator or a prosthetic group carrying a radioisotope for nuclear imaging directly to the aptamer 88. Recent developments of radiolabeled aptamers and aptamer-based NPs for nuclear imaging are discussed in the following paragraphs.

Since 2018 almost no studies used gamma emitting isotopes for SPECT imaging of aptamer-based probes, with the exception of a report of MUC1 targeted, PEG-modified PAMAM dendrimers that were labeled with ^67^Ga-DTPA 89. A biodistribution study in MDC-7 tumor bearing rats revealed high tumor uptake of 5.11 ± 1.22, 18.37 ± 3.59 and 9.17 ± 1.19% ID/g at 6, 24 and 48, respectively. SPECT imaging confirmed clear tumor accumulation at 24 and 48 h, with low background signal. Regrettably, no image quantification was performed, and the biodistribution and imaging study lacked control experiments with untargeted radiotracers, which makes it difficult to compare the targeting effect of the MUC1 aptamer to other studies.

Several probes used positron emitting radionuclides for PET imaging. Fletcher *et al.* 90 reported a ^89^Zr-labeled hyperbranched PEG-polymer (HBP) targeted with the anti VEGF-A locked nucleic acid aptamer RNV66, which was originally described in 2015 91. The deferoxamine-functionalized nanocarrier with a molecular weight of approximately 50 kDa and a hydrodynamic diameter of 7.2 nm was labeled with ^89^Zr in quantitative yield and serum stability of 85% and 62% was observed *in vitro* after 3 and 14 days, respectively*.* After injection of 1-2 MBq of aptamer-targeted or non-targeted nanocarrier into MDA-MB-231 xenograft bearing mice, PET/CT images were acquired at 0 h and 1, 2, 3, 6 and 9 days. Pharmacokinetics and organ activity were estimated by ROI analysis of relevant organs. The blood elimination half-life of the aptamer targeted HBP was 13.4 h, while no circulation half-life of untargeted HBP was reported. Tumor accumulation for the aptamer targeted HBP peaked at day 3 with approximately 4% ID/g at which time the untargeted HBP reached <2% ID/g. Interestingly, a similar trend is observable also in heart, kidneys, spleen and especially liver, which shows substantially higher organ uptake for aptamer targeted HBP compared to untargeted HBP. The authors further incorporated Cy5.5 into the polymer and found that the aptamer targeted HBP accumulated in the tumor periphery.

**Figure 3:**
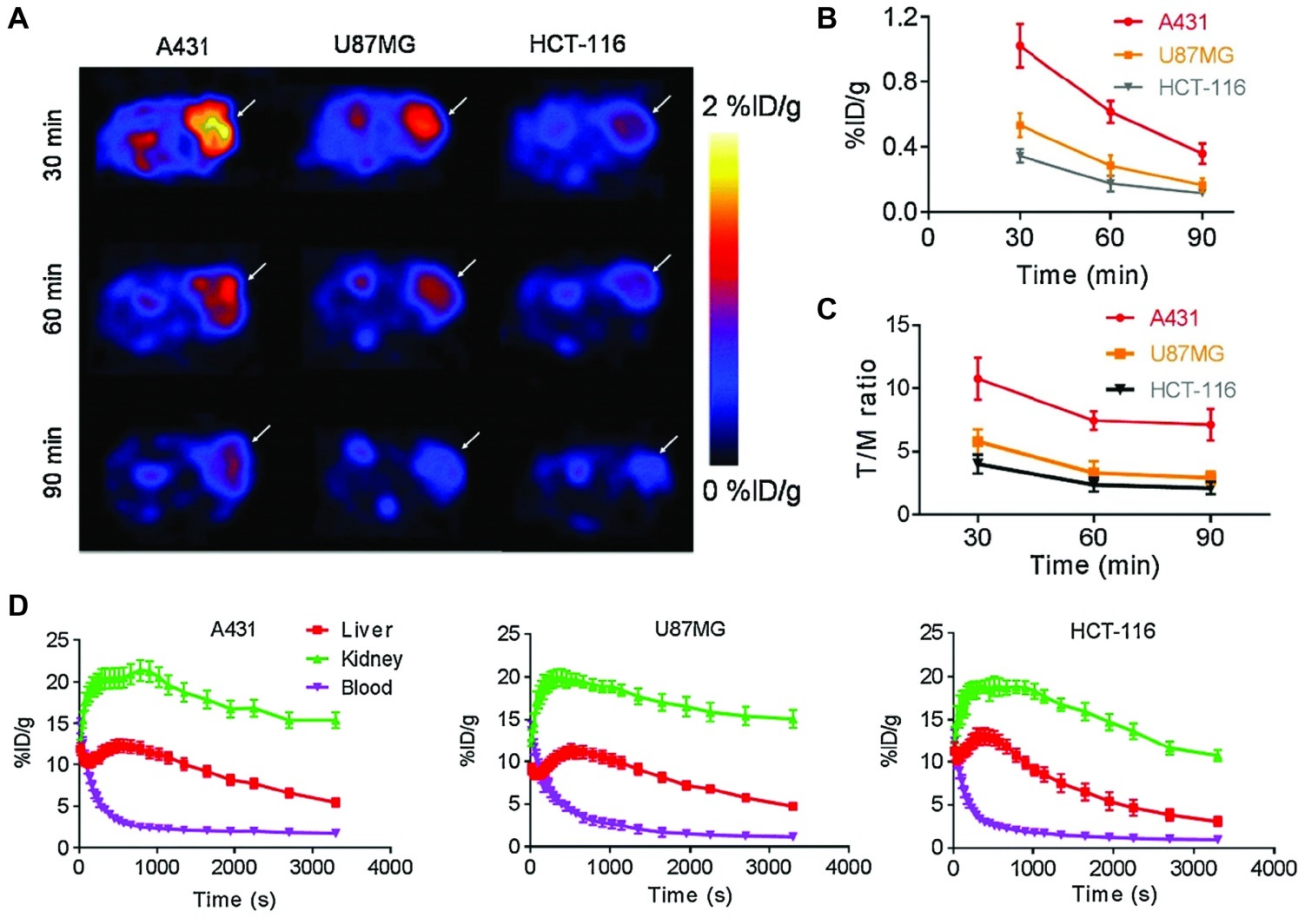
PET imaging of ^18^F-labeled S6 aptamer in vivo and quantification of tumor uptake and pharmacokinetics. (A) In vivo PET images of radiolabeled S6 aptamer in A431, U87MG and HCT-116 xenografts (high, medium and no EGFR expression, respectively). (B) ROI-based image analysis to determine tumor uptake of S6. (C) Tumor to muscle ratio of S6. (D) Dynamic PET imaging to determine pharmacokinetics in different xenograft models, highlighting the rapid blood clearance of aptamers. Adapted with permission from 92. Copyright 2018 Springer Nature.

Cheng *et al.* used a 2'F-stabilized and 5'-alkynated ME07 RNA aptamer for ^18^F PET imaging of three EGFR expressing tumor xenograft models 92. Labeling was performed *via* Cu-catalyzed azide-alkyne cycloaddition with [18F]fluorobenzoyl-azide in 70±4% radiochemical yield. Flow cytometry and confocal microscopy demonstrated cell staining correlating to high, medium and no EGFR expression levels of A431, U87MG and HCT-116 cells, respectively. The same correlation was found in tumor bearing mice injected with 3.70-5.55 MBq of ^18^F-labeled aptamer. While the overall tumor accumulation is modest with less than 1% ID/g based on ROI analysis even for A431 tumors, the tumor-to-blood and tumor-to-muscle ratios were favorable and showed significant higher values for A431 tumors both by image-based ROI analysis and *ex vivo* biodistribution. Ninety minutes after injection the T/B and T/M ratios amount to 3.89, 1.76, 1.46 and 8.65, 3.71, 2.12 for A431, U87MG and HCT-116, respectively. Dynamic PET imaging over 60 min showed peak tumor uptake of 3.24, 2.17 and 1.35% ID/g at 3 min post injection in A431, U87MG and HCT-116 cells, respectively, followed by rapid decrease (Figure 3). Despite the 2'F-modification, the tracer shows relatively modest *in vivo* stability in healthy mice, with intact aptamer decreasing from 42.60% to 14.98% after 5 and 20 min circulation, respectively.

Another DNA aptamer, SH-1194-35 was labeled with ^18^F *via* amide coupling with N-succinimidyl 4-[^18^F]fluorobenzoate for PET imaging of HER2 expressing tumor xenografts 51. Mice bearing HER2-positive BT474 or HER2-negative MDA-MB-231 xenografts in the axilla were intravenously injected with 13.7±1.1 MBq of labeled antibody and imaged dynamically for 30 min followed by static imaging at 60, 90 and 120 min post injection. Regrettably, only images at 60 min are shown and no further pharmacokinetic characterization was performed. ROI based analysis at 60 min revealed a tumor accumulation of 0.62±0.04% ID/g in HER2-positive xenografts while HER2 negative animals had a small but statistically significant lower tumor uptake. Biodistributions of BT474 tumor bearing mice after 30 min showed similar results, but no results were shown for the control group. Interestingly the tumor uptake of the anti HER2 aptamer at 60 min was comparable to the anti-EGFR aptamer in a study by Cheng *et al.* 92, although differences in study design and analysis make a direct comparison difficult.

Ozerskaya *et al*. took an interesting approach for ^11^C labeling of AS-14 aptamer for PET imaging of Ehrlich ascites carcinoma^93^. Instead of directly labeling the aptamer, a ^11^C-labeled complementary primer was hybridized to AS-14. The labeled primer was synthesized from cyclotron produced ^11^C with 42% radiochemical yield. Metastatic tumors were created by injection of Ehrlich ascites carcinoma cells into the tail vein of mice, followed 9 to 16 days later by the injection of 10 MBq [^11^C]AS-14. Highest contrast was achieved after 40 min and even small metastatic lesions in heart, lungs, intestines and ribs could be detected with high accuracy, confirmed by histology. Interestingly, [^11^C]CH_3_-AS-14 was able to detect metastatic lesions better than ^18^FDG, while control experiments with ^11^C labeled scrambled sequence or just the primer were not able to detect metastatic lesions.

PEGylation of aptamers is a formulation strategy that improves the pharmacokinetic properties by reducing renal elimination and enzymatic degradation 94. In an effort to capitalize on the increased circulation half-life Li *et al.* used the PEGylated anti-EpCAM DNA aptamer, #1-F, tethered to DOTA for labeling with ^64^Cu and PET imaging of MDA-MB-231 bearing xenografts 95. In contrast to the aforementioned labeling strategies with ^18^F, the aptamer was directly labeled through single-step chelation of ^64^Cu in 80-90% radiochemical yield with >98% radiochemical purity. ROI-based tracer uptake in the tumor peaked at 24 h with 2.1% ID/g. Biodistributions of EpCAM-negative U937 and positive MDA-MB-231 tumors at 24 h confirmed tumor specific uptake with 0.75 and 2.4% ID/g, respectively. Furthermore, co-injection of 25-fold excess of unlabeled aptamer resulted in complete blocking of specific binding in MDA-MB-231 tumor animals. With a tumor retention of up to 24 h this result is a strong testament to the improved plasma circulation time of PEGylated aptamers.

Another strategy to overcome the poor plasma stability of aptamers was reported by Xia *et al*. who synthesized sgc8-targeted gold NP nanoclusters through simple electrostatic interaction 96. To synthesize the nanoclusters, sgc8 was mixed with gold NPs in 14:1 ratio, which led to a particle size of 94 nm. Flow cytometry showed an improved K_d_ of 3.11 nM for the nanoclusters, compared to 4.57 nM for free sgc8 aptamer. Importantly, in the assembly of sgc8 in gold nanoclusters, the aptamer could still be detected after 48 h of incubation in 20% FBS, whereas free sgc8 was completely degraded after 12 h. *In vivo*, this was reflected by a plasma half-life of 2.15 h for ^68^Ga-labeled sgc8-gold nanoclusters, compared to 1.83 h for free ^67^Ga-sgc8. PET/CT imaging was performed in mice bearing HCT-116 xenografts and showed a tumor to muscle ratio of 16.6 for the targeted gold nanoclusters after 1 h and a 9-fold higher tumor to muscle ratio compared to directly labeled sgc8 after 2 h.

The fast enzymatic degradation and excretion of RNA and DNA *in vivo* is without doubt one of the main contributing factors for the small number of papers in the recent years using directly labeled aptamers for nuclear imaging 97. However, formulation strategies that increase size, such as PEGylation or conjugation to carrier molecules could lead to the development of a new generation of aptamer-based imaging probes with improved pharmacokinetics.

### 3.3 SERS

Raman scattering occurs when small fractions of incident photons (approximately 1 in 10^7^) interact with matter through inelastic scattering, resulting in an energy exchange of incident light and the scattering material. Through Raman scattering, the emitted photons have altered frequency and wavelength, depending on the material properties of the scattering material. The unique nature of the scattering properties for different Raman reporter molecules confers a high degree of specificity to this spectroscopic technique and the generated signal is characterized by narrow emission bands of around 2 nm. The unique “Raman fingerprint” of different reporters is especially interesting for multiplexed applications where a multitude of reporter molecules can be detected in parallel. An inherent limitation for* in vivo* applications, however, is the low fraction of incident photons that undergo Raman scattering. The discovery of SERS in 1973, which enhances the Raman intensity of a molecule up to a factor of 10^14^ to 10^15^ allows the detection of single molecules, outcompeting even single-molecule fluorescence reporters 98. SERS is a phenomenon that occurs when Raman reporter molecules are absorbed to the surface of metal surfaces or nanomaterials 99. Although the exact mechanism is still under debate, it is recognized that a combination of electromagnetic - most notably through the generation of surface plasmons - and chemical effects contribute to the overall signal enhancement 100. An in-depth review of mechanisms that contribute to the signal enhancement in SERS falls beyond the scope of this review, but excellent reference literature is available for consultation 101. The use of SERS for *in vivo* applications is a relatively novel concept and the first use of SERS nanoprobes for detection of subcutaneous tumors emerged in 2008, pioneered by Shuming Nie at the Georgia Institute of Technology 102 and Sanjiv Sam Gambhir in Stanford 103. While only a few previous studies of aptamer-based SERS probes in mice have been reported to date, the most important is a proof-of-principle study by Pal *et al.* from 2017 using MUC1 aptamer targeted SERS probes in murine breast cancer models 104. As in fluorescence imaging, limited tissue penetration depth remains a challenge for *in vivo* applications and potential clinical translation in human patients 105. In this chapter we present recent aptamer-based SERS probes for* in vivo* tumor detection in mice.

Xinjing Tang *et al.* previously described an AS1411-targeted SERS nanoprobe with three different biorthogonal Raman report molecules for multiplexed detection of tumor cells 106. Due to a lack of *in vivo* stability of these initial probes, gold NPs were modified with a novel Raman reporter molecule with a Raman shift of 2205 cm^-1^ through a more robust C-Au linkage and further modified with AS1411 or MUC1 aptamer, in order to create a SERS probe with improved physicochemical properties for *in vivo* targeting. Mice with MCF-7 tumor xenografts were intravenously injected with 0.6 nmol of the SERS nanoprobe and images of the tumor region taken 4 h later with a slit scanning Raman microscope. Both AS1411 and MUC1 targeted nanoprobes were clearly detected in the tumor while no signal was observed for non-targeted nanoprobes 107. As an evolution of this design, they created a multiplexed cocktail of AS1411-, RGD peptide- and anti-CD44 antibody targeted SERS probes with distinct Raman reporter functionalities (azide (2120 cm^-1^), diynyl (2205 cm^-1^) and cyano (2230 cm^-1^), respectively). To improve the stability of the nanoprobes in biological media, the surface was further coated with silica and PEG, leading to a 3-fold higher tumor accumulation compared to unprotected sensors. The SERS cocktail was able to bind MDA-MB-231 and MCF-7 cells, with distinct Raman peaks for the different sensors and revealed a lower CD44 expression profile in MCF-7 cells. This finding was substantiated by imaging of tumor bearing mice with a slit scanning Raman microscope after intravenous injection of the multiplexed cocktail. No signal was observed for non-targeted probes or in the surrounding healthy tissue 108. This proof-of-principle study paves the way for possible cancer phenotyping using spectroscopically distinct Raman reporters and further demonstrates the broad range of possible targeting moieties, including aptamers.

In order to capitalize on the photothermal capability of the gold nanocarrier, another study investigated the theranostic use of aptamer-targeted gold nanorods for photothermal therapy (PTT) and SERS imaging 109. Here the diynyl reporter was conjugated to the surface of the gold nanorods *via* a double S-Au bond through a lipoic acid linker. Compared to their unspecific control sequences, a 43-fold and 32-fold higher tumor accumulation of AS1411 and MUC1 targeted nanoprobes was measured in MCF-7 tumor xenografts 6 h post intravenous injection. Furthermore, complete tumor growth inhibition was achieved with a single near infrared irradiation of tumor regions for the aptamer targeted nanorods after allowing them to circulate for 6 h, while untargeted controls did not show any temperature increase in the tumor area and no growth inhibition. This impressive result clearly demonstrates the potential for simultaneous tumor detection and PTT with this theranostic platform, although it should be noted that imaging was only performed in 50 x 50 µm^2^ sections of the tumor area.

**Figure 4:**
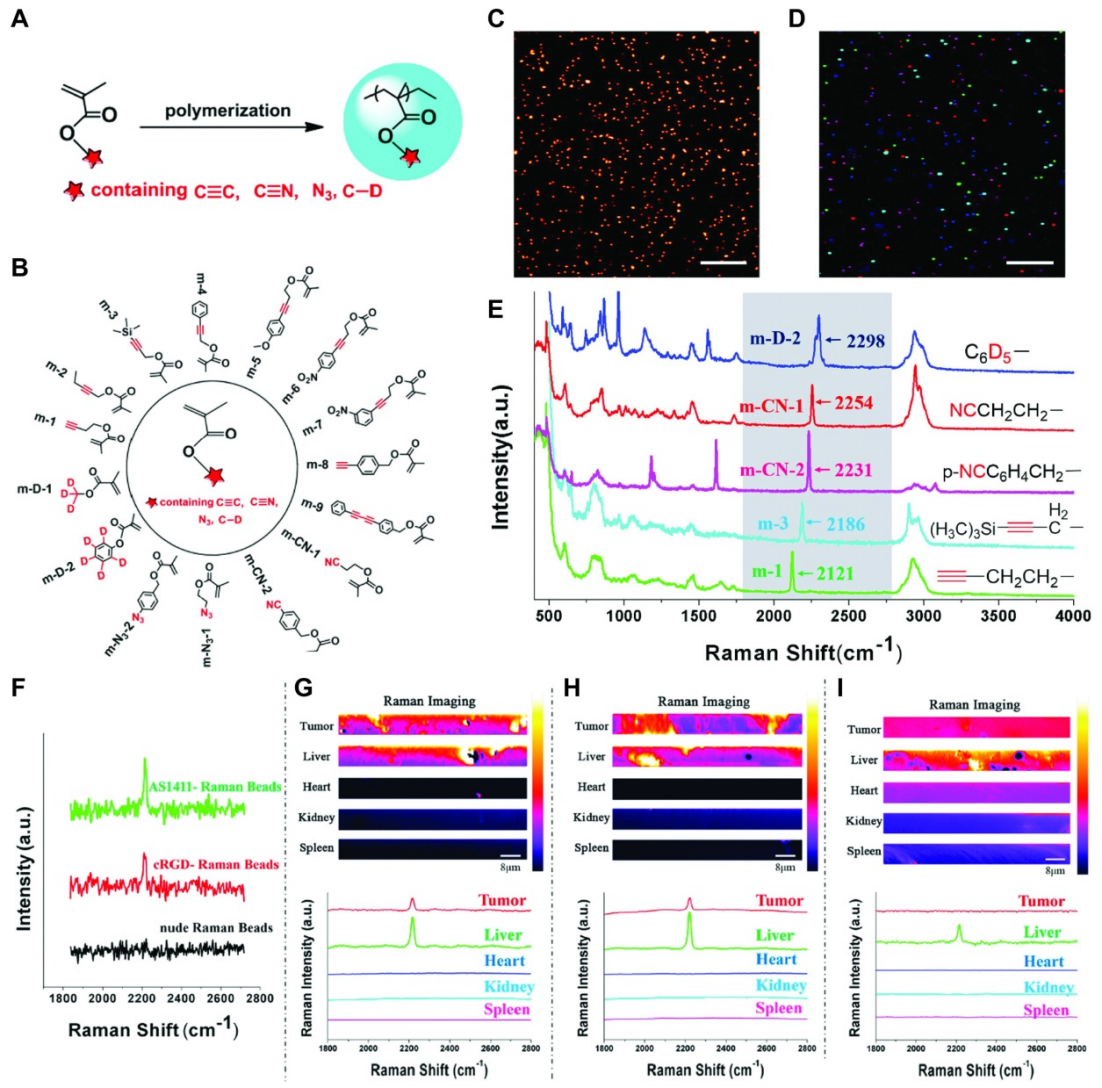
Polymethacrylate beads for spontaneous Raman scattering (SRS). (A) Polymerization of functionalized poly(methacrylate) monomers to generate a library of (B) 15 distinct Raman reporters. (C) Representative SRS imaging of m-CN-2. (D) SRS imaging of five mixed Raman beads. (E) Raman spectra of five mixed Raman beads with Raman silent region shown in grey box. (F) In vivo SRS imaging of m-9 with AS1411 and cRGD targeting or untargeted beads (G), (H), (I) ex vivo organ scanning of AS1411-m9, cRGD-m9 and untargeted m-9, respectively. Adapted with permission from 110. Copyright 2019 American Chemical Society.

Alongside the prolific work with gold NP SERS reporters, another type of Raman probe that works through concentration dependent spontaneous Raman emission was explored. To this end, poly(methacrylate) beads with high densities of alkyne, nitrile, azido, and carbon-deuterium moieties were synthesized to generate a library of 15 Raman reporters with distinct shifts in the Raman silent region (1800 - 2500 cm^-1^) 110. The paper presents intriguing *in vitro* results for cell (co-)staining with five different reporter NPs targeted with either AS1411, MUC1 aptamer or the cyclic arginine-glycine-aspartic acid (cRGDfk) peptide targeting α_v_β_3_ integrin and untargeted beads.* In vivo* tumor detection was performed analogous to the experiments with gold NP-based SERS reporters to reveal accumulation of AS1411-, MUC1- and cRGDfk-targeted beads in tumors, while no signal was observed for untargeted beads (Figure 4). *Ex vivo* organ sections further confirmed that apart from the liver no uptake of targeted or untargeted beads was detectable in heart, kidneys, or spleen.

SERS imaging of larger surfaces or organisms remains a technical challenge, despite recent developments in this area that made organ scanning 111 and even imaging of small animals principally feasible 112. Presently,* in vivo* Raman imaging is still lagging behind more established molecular imaging modalities like PET, MRI and fluorescence imaging. However, the here presented nanoprobes present important advancements in the field that could one day bridge the gap from more *in vitro* focused biosensing applications towards molecular imaging. Due to their ability to recognize molecular motifs with high specificity and affinity, aptamers are ideally suited to create targeted SERS nanoprobes for theranostic applications 109, 113.

### 3.4 US

Ultrasound imaging or echography is a non-invasive method that uses ultrasound pulses (>20'000 Hz) that are generated from a probe and reflected from tissue interfaces. The time that passes for the reflected ultrasound waves to return to the probe can be used to reconstruct images containing anatomical information about the probed tissue or functional information about blood movement. Ultrasound is the most used medical imaging modality in clinical practice by number of scans performed annually 114. Ultrasound contrast agents are micron- or nano-sized bubbles encapsulated in a thin layer of lipids, albumin or other materials like multi-walled carbon nanotubes. The bubbles generally contain an inert gas with high molecular weight and low solubility in water, such as perfluorocarbons or sulfur hexafluoride. Due to their size and lower density compared to blood, US contrast agents strongly scatter the soundwaves. In addition, the bubbles are deformed resulting in broadband acoustic response compared to the more uniform echo of surrounding tissue 115. Generally, US contrast agents provide information about blood flow in vessels or organs and can be used for identification and classification of neoplasms. Targeted US contrast agents bind to a specific biomarker *via* peptides, proteins, antibodies or aptamers and are able to accumulate at the target site through ligand-receptor adhesion, which can provide additional information for conditions such as inflammation, thrombosis and cancer 116. In the following paragraph recent developments of aptamer targeted US contrast agents are discussed.

Zhu *et al.* selected a novel DNA aptamer against carbonic anhydrase IX (CAIX) for the preparation of aptamer-targeted nanobubbles for US contrast enhanced tumor imaging 57. CAIX is an enzyme that is crucial for intracellular pH maintenance and is overexpressed in a broad range of tumors, including renal, cervical, colon, prostate and breast cancer. The thiol modified aptamer was conjugated to perfluoropropane-filled lipid nanobubbles with a size of approximately 500 nm *via* maleimide coupling. US imaging of intravenously injected nanobubbles functionalized with scrambled ssDNA into CAIX-positive 786-O and HeLa or CAIX-negative BxPC-3 xenografts showed comparable tumor contrast enhancement in all tumors, while aptamer targeted nanobubbles led to a moderate but statistically significant further increase in signal intensity only in 786-O and HeLa xenografts.

AS-1411 functionalized lipid nanobubbles were used for contrast enhanced US imaging of triple-negative breast cancer (TNBC) 117. *In vitro*, the perfluoropropane filled bubbles were able to selectively bind MDA-MB-231 and MDA-MB-468 tumor cells while no binding was observed to normal kidney HK-2 cells. Imaging was performed in TNBC tumor bearing mice. For 10 min, the targeted nanobubbles showed an increased signal intensity compared to non-targeted bubbles in MDA-MB-231 and MDA-MB-468 tumor models. While peak intensity and time to peak intensity were not different between groups, targeted nanobubbles showed a statistically increased area under the curve, suggesting their potential use as TNBC specific US contrast enhancing agents.

Gu *et al.* constructed anti-PSMA targeted multi-walled carbon nanotubes for contrast enhanced US imaging of prostate cancer 118. The 300 nm long and 15 nm wide tubes were PEGylated and covalently modified with A10-3.2 RNA aptamer that specifically binds PSMA with low nanomolar affinity 119. US imaging in mice bearing PC-3 tumor xenografts with untargeted and targeted multi-walled carbon nanotubes showed strong contrast enhancement of tumor tissue within 1 h of injection for both groups, reaching maximum intensity at 8 h and gradual decrease thereafter. Contrast enhancement was also observed in kidneys, but not in the heart. The results of the study remain qualitative and additional questions regarding long term toxicity and biocompatibility of the multi-walled carbon nanotubes remain.

### 3.5 CT

X-ray computed tomography (CT) or conventional 2D radiography is a commonly used imaging technique that generates highly resolved images of biological specimen through attenuation of high energy electromagnetic radiation by different tissue composition and density. Generally, higher atomic number and density leads to increased contrast, which can be exploited to generate CT contrast agents with attenuation factors several orders of magnitude higher than biological tissue. Typical CT contrast agents are composed of iodinated compounds, metal chelates, and nanomaterials 120. Noteworthy are also gold NPs due to their ideal physicochemical suitability for X-ray attenuation and good biocompatibility which can be problematic for other classes of CT contrast agents 121. Interestingly, no aptamer-based pure CT imaging probes have been reported recently, although two novel theranostic probes are discussed below.

Cao *et al.* developed a theranostic platform that greatly increased CT contrast and effectively neutralized tumor cells upon sonodynamic stimulation 122. The nanoprobe is comprised of TiO_2_ nanosheets functionalized with AS1411 aptamer and triphenylphosphine for mitochondria targeting. Additionally, gold NPs were selectively grown on the edge of the 40 nm sized sheets for CT contrast enhancement and further increased reactive oxygen species (ROS) generation. The probe was able to specifically bind to MCF-7 cells *in vitro* and exhibited no toxicity up to a concentration of 200 µg/mL. Tumor accumulation of the aptamer/triphenylphosphine targeted nanosheets in MCF-7 xenograft mice amounted to approximately 5% ID/g determined by ICP-MS and strong accumulation in liver and kidney was observed. CT images of mice injected with the nanoprobe showed clear accumulation in the tumor, although the experimental conditions are not described in enough detail to explain the complete absence of contrast in excretory organs, as would be expected based on the biodistribution results. Sonodynamic therapy resulted in complete tumor regression in mice treated with the targeted nanoprobe, while untargeted nanosheets had a reduced effect.

Alibolandi *et al.* developed MUC1 aptamer targeted PAMAM dendrimers loaded with curcumin and gold nanocrystals as a theranostic platform for treatment and CT contrast enhanced imaging of colon adenocarcinoma 123. The formulation was optimized for prolonged duration of action, as the aptamer was 3'-modified with inverted dT nucleotides to enhance the enzymatic resistance. Confocal laser scanning microscopy revealed preferential and partially blockable uptake in HT29 and C26 cells compared to CHO cells, although the lack of quantitative analysis prevents direct comparison. Even though flow cytometry revealed enhanced uptake of targeted over the non-targeted NPs, free curcumin showed the highest uptake for both HT29 and CHO. The theranostic formulation demonstrated almost complete inhibition of tumor growth in C26 xenograft mice over a period of 30 days, after intravenous administration twice per week during the first 21 days at a curcumin dose of 2 mg/kg. Reduced efficacy was observed for the untargeted formulation. Qualitative CT images 12 h p.i. showed clear contrast enhancement in the tumor region and a reduced contrast for non-targeted NPs.

## 4. Fluorescence Imaging

*In vivo* fluorescence imaging is a non-invasive technique to evaluate the pharmacokinetic behavior and biodistribution of a (bio)-molecule or NP of interest that requires nothing more than a fluorophore with a sufficiently long excitation/emission wavelength for tissue penetration. Components of a nanoformulation can have inherent fluorescent properties (e.g., doxorubicin (DOX) or quantum dots), or dedicated fluorescent dyes like cyanines, Alexa Fluor^TM^ and DyLight^TM^ can be added to create fluorescent probes. An inherent disadvantage of fluorescent imaging is the low tissue penetration depth due to scattering and attenuation of emitted light in biological tissue as well as backscattering of incident photons 124. This can be improved by using near-infrared fluorophores with excitation and emission in the window of 650 to 1350 nm, where tissue penetration depth of light is maximal and autofluorescence is low 125. Due to this limitation fluorescent imaging in humans has largely been confined to perfusion studies of body parts 126 and fluorescence guided surgery 127. In the preclinical setting however, fluorescence and near-infrared imaging are useful and frequently used for the development of nanomedicines, drug delivery systems and diagnostic probes.

The often-quoted aptitude of aptamers for chemical modifications makes them especially well-suited for conjugation of fluorophores. A growing number of companies offers now chemical modifications to aptamers with fluorophores, but also quenchers, functional groups, linkers, and modified nucleobases. Also, standard *in vitro* characterization techniques like flow cytometry, confocal microscopy and immunohistochemistry require fluorophores. It is therefore not surprising to see fluorescence imaging as the most prevalent imaging modality in preclinical research, especially for the early evaluation of novel aptamers. Nevertheless, as exemplified by the fluorescently labeled aptamers without further formulation strategies in section 2, it is unlikely that they become successful clinical imaging probes due to the unfavorable pharmacokinetics and fast degradation of aptamers *in vivo* 128. To address these shortcomings, aptamer-based NP formulations and the development of “smart” aptamer probes are a promising avenue towards the realization of aptamers for diagnostic or theranostic purposes. In the following paragraphs, recent developments of NP-based probes for fluorescence or multimodal imaging and efforts to create more sophisticated “smart” fluorescent imaging probes are discussed.

### 4.1 Nanoparticle Based Fluorescent Imaging Probes

An EGFR targeting nanocomposite for multimodal fluorescence and MRI imaging based on a DNA nanotriangle carrier was described by Song *et al.* The imaging probe is composed of a PEG_5k_-decorated, self-assembling DNA polyhedron, functionalized with DOTA-Gd for MRI imaging and DyLight 800 for fluorescent imaging 129. For targeting, the anti-EGFR RNA aptamer CL4, originally described in 2011 was used 130. *In vivo*, the nanocomplex showed moderate but statistically significant higher tumor uptake compared to untargeted, non-PEGylated, and untargeted/non-PEGylated control groups at 24 h post injection. Interestingly, MRI imaging showed clearly delineated tumor uptake of the targeted formulation as early as 1 h after injection, with peak signal at 2 h. In contrast, the clinically used contrast agent Gd-DOTA showed weaker contrast enhancement over the same period.

Albumin has long been recognized to substantially increase the plasma half-life of a variety of non-covalently absorbed biomolecules due to its large size and long circulation half-life of 19 days 131. In an effort to capitalize on this “albumin binder” effect for improving biological half-life and tumor accumulation of aptamers, Weihong Tan *et al.* applied a “hitchhiking strategy” by modifying sgc8 with the dye Evans Blue (EB), which binds to hydrophobic regions of human serum albumin 132. *In vitro* this EB-modified sgc8 not only had a significantly increased stability in cell medium supplemented with 20% FBS or human serum, but also showed a more than 2-fold increased binding affinity to HCT-116 cells. While binding affinity of EB-sgc8 was comparable to unmodified sgc8, a reduction in specificity was observed. *In vivo* and *ex vivo* fluorescence imaging of Cy5-modified sgc8 and control ssDNA in HCT-116 xenografts showed the expected fast renal clearance for both sequences and a marginally higher tumor uptake of sgc8 in the first hour. In contrast, EB-modified sequences showed increased tumor uptake and slower excretion, attributable to the EPR effect of the nanoconjugates that exceeded the renal clearance threshold of 30 to 50 kDa 133. The delivery efficiency of 19.37 and 11.45% for EB-sgc8 and EB-library compared to 7.2 and 6.3% for the unmodified sequences, respectively, is an encouraging testimony for this simple and potentially universally applicable strategy to improve the notoriously poor pharmacokinetics of aptamers. Nevertheless, more research with other cell lines is needed to address the observed reduction in binding specificity for this targeting strategy.

Using the cholecystokinin-B (CCK-B) receptor specific DNA aptamer AP1153 134 for targeting, Abraham *et al*. synthesized ICG encapsulated calcium phosphosilicate NPs as a simple probe for fluorescence imaging of pancreatic tumors 135. No appreciable organ uptake other than in the tumor was observed in PANC-1 xenograft bearing mice, which is in stark contrast to the usual high liver and kidney uptake of other NP formulations. Tumor uptake was first observed after 12 h and peak signal intensity occurred after 18 h. In contrast, no uptake was observed for non-targeted NPs. Further *ex vivo* studies confirmed that targeted NPs accumulated also in PC-3 prostate xenografts and were able to extravasate evenly throughout tumor tissues. Furthermore, CCK receptor blockage with proglumide reduced tumor uptake, confirming the role of aptamer-mediated targeting. Given the minimal off-target binding, further evaluation of this probe is warranted.

### 4.2 Smart Imaging Probes

In contrast to the aforementioned “always on” probes that rely on active or passive accumulation in the tumor, several groups have explored strategies to reduce off-target binding or reduce background signal by developing probes that are designed to exhibit fluorescence only under specific conditions. Although these “smart” probes could potentially alleviate some of the general drawbacks of fluorescent probes, namely high background fluorescence and low specificity, the increased complexity arguably complicates clinical translation.

A potential way to exploit the generally acidic tumor microenvironment (TME) for molecular imaging, are pH responsive aptamer probes. Two different approaches in the development of such probes were taken by Kemin Wang *et al*. The first probe is composed of a fluorescently labeled aptamer that is hybridized to a split i-motif functionalized with a quencher, which renders the probe non-fluorescent at physiological pH 136. In acidic environments of pH <6.8 the i-motif dissociates from the strand to form an intramolecularly stabilized structure, restoring the fluorescence signal of the labeled aptamer. Due to the fact that the hybridizing portion of aptamer and split i-motif is a generic linker, this activation strategy could theoretically be employed for a range of different aptamers. This was demonstrated *in vitro* by confocal microscopy of the split i-motif pH sensor (pH-AAP) with sgc8, AS1411, and TD-05 aptamers. To verify the applicability of the system *in vivo*, mice bearing subcutaneous SMMC-7721 or CCRF-CEM tumors were intratumorally injected with pH-AAP-ZY11 and pH-AAP-sgc8c, respectively. The same probes were intramuscularly injected on the contralateral side to compare the pH response in normal tissue. pH-AAP-sgc8 showed a rapid onset of fluorescence in the tumor within 1 minute, while the intramuscular injection was only weakly fluorescent. In comparison, the intratumoral fluorescence increase of pH-AAP-ZY11 was slower and less pronounced. Due to the preliminary nature of the study, it remains uncertain how much of the fluorescence difference is attributable to the difference in the tumor models and imaging probes. Nevertheless, this potentially generalizable pH responsive probe could be used as a universal probe for molecular imaging of tumors.

**Figure 5:**
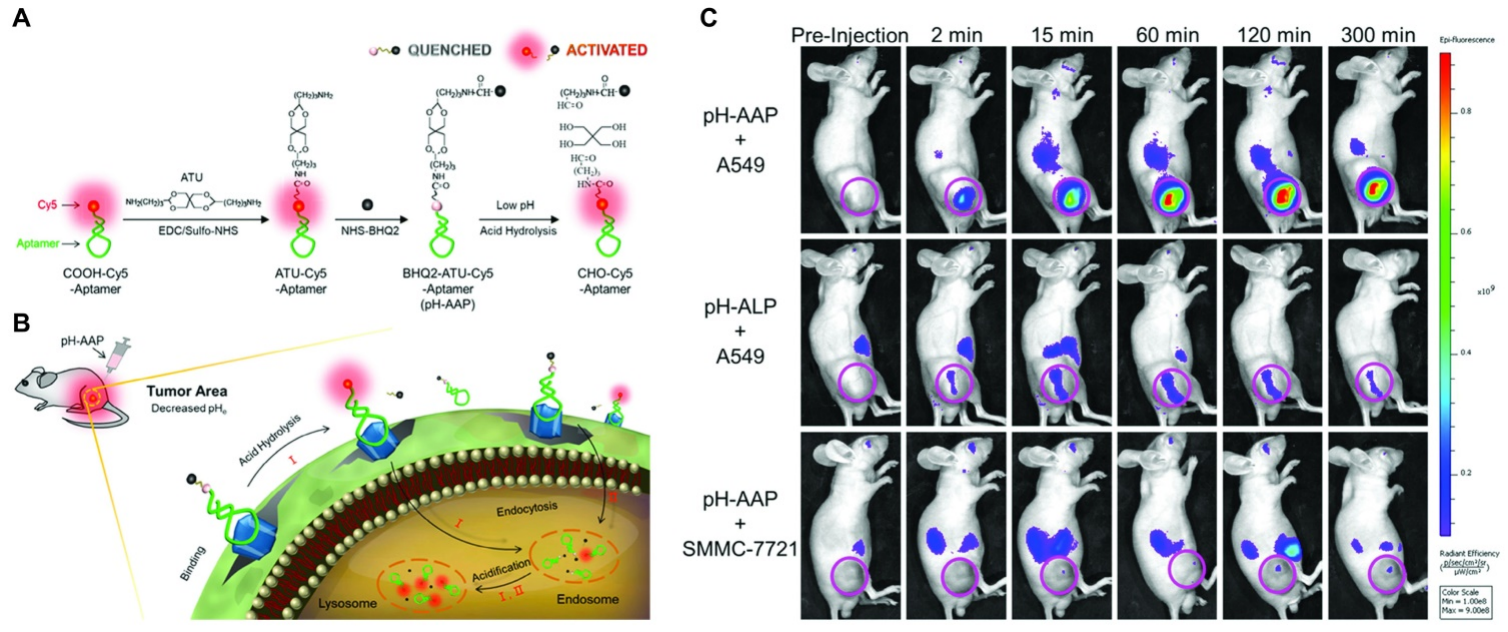
Bispecific tumor imaging using a pH-activatable fluorescent aptamer. (A) Construction of pH-activatable aptamer probe (pH-AAP) and its acid-responsive mechanism. (B) Schematic overview of aptamer binding and acid-responsive cleavage of pH-AAP for contrast-enhanced bispecific tumor imaging. (C) In vivo fluorescence images of pH-AAP in mice bearing A549 xenografts (top row), a non-binding version of the aptamer (middle row) and pH-AAP in a non-target xenograft model. Adapted with permission from 138. Copyright 2019 American Chemical Society.

In the second approach, a probe containing a pH-cleavable linker was developed to improve the often unsatisfactory noise suppression and poor stability of split activatable aptamer probes (SAAPs). The probe consists of Cy-5-modified S6, a DNA aptamer specific for A549 cells 137, linked to the quencher BHQ2 *via* the acid cleavable acetal linker 3,9-bis(3-aminopropyl)-2,4,8,10-tetraoxaspiro [5.5]undecane (ATU) 138. Initially, the fluorophore's proximity to the quencher leads to fluorescence suppression of 98%. *In vitro* this activatable aptamer probe (pH-AAP) showed superior quenching in neutral buffer over longer periods compared to a variant of S6 in which quenching was achieved by intramolecular hybridization similar to other SAAPs. Compared to the SAAP control and directly labeled S6, the acid cleavable probe showed markedly lower background fluorescence in normal mice over the course of 60 min owing to the covalent link between fluorophore and quencher. Mice bearing A549 tumors showed rapid tumor uptake within 2 min of administration, with high levels of fluorescence detectable up to 300 min. Most notably, non-specific fluorescence was only detectable in the kidneys and off-target fluorescence was extremely low for both a scrambled version of the probe (pH-ALP) and the probe in non-target SMMC-7721 cells (Figure 5). These results present an impressively long-lasting tumor signal with extremely low background noise and could be an important step towards other fluorescent imaging probes using this signal activation strategy.

By exploiting the hypoxic TME, Zhou *et al.* explored an approach to reduce nonspecific binding of aptamers to normal tissue, which is a common issue for tumor antigens that are over- but not exclusively expressed by tumors. Taking inspiration from an azobenzene-PEG caged cell penetrating peptide that was first described in 2014 139, a similar caging strategy was developed by conjugating an azobenzene-PEG_5k_ moiety to transferrin receptor 1 specific XQ-2d aptamer 140. This sterically demanding moiety prevents binding of the aptamer to the target protein in physiological conditions, while hypoxic conditions in the TME promote reductive cleavage of the azobenzene linker. In DU145 xenografts, caged XQ-2d was taken up into tumors within 30 min and was detectable up to 2 h. Uncaged XQ-2d showed a similar tumor uptake profile. Control aptamers or permanently caged XQ-2d, however, were not visible in the tumor anymore after 2 h. Although the proposed mechanism of uncaging in hypoxic tumor environments seems to restore target recognition of the aptamer, no obvious difference in off-target fluorescence between free and caged XQ-2d was detectable, and no quantitative image analysis was performed. The high fluorescence signal in kidneys and liver due to the characteristic elimination of aptamers and the generally poor spatial resolution of *in vivo* fluorescence imaging is a considerable obstacle for diagnostic imaging with aptamers. It remains to be seen if this conceptually intriguing caging strategy of aptamers can be used to create probes with better contrast and less background imaging.

## 5. Aptamers for Theranostic Applications

“Theranostics” is a term that describes agents that combine both therapeutic and diagnostic capabilities in a single probe. For personalized medicine, especially in oncology, molecular imaging can identify the precise location and biomolecular nature of a malignancy in order to decide on the best treatment modality. Similarly, imaging can be used to monitor if a treatment has the desired effect, i.e., shrinking of tumor mass takes place or metastatic nodules are reached by the therapy. The advantage of theranostic probes is that these traditionally separate roles can be combined into a single probe which reduces the burden on patients as well as health-care providers. Virtually all theranostic probes require a targeting agent against a specific molecular marker to confer specificity and reduce side effects to normal tissue. Since aptamers with high specificity can be selected against a variety of targets and even whole cells with abnormal phenotype, they are ideal targeting moieties for theranostic agents. Theranostics strongly benefit from nanomaterials due to their ability to serve as drug carrier, imaging reporter or intrinsically therapeutic agent. It is therefore not surprising that the combination of nanomaterials and aptamers dates back to the very dawn of theranostics as a technology 147. In the following paragraphs we present recent studies that use aptamers as targeting moiety for nanomaterials as well as novel aptamers that possess innate biological activity and have been used for molecular imaging *in vivo*. We focus on studies with a clear interest of *in vivo* molecular imaging and did not include reports where therapy was the only goal of the study.

### 5.1 Aptamers with Therapeutic Effect

A particularly interesting class of theranostic agents are aptamers with intrinsic therapeutic effect. Since aptamers are able to specifically bind molecular targets such as receptors, kinases, or other enzymes it is not surprising that aptamers have been explored early on for potential therapeutic effects such as anticoagulation, anti-inflammation and especially for growth inhibition in cancer therapy 14. A prominent example for this class is AS1411, a guanosine-rich oligonucleotide (GRO), which binds to cell surface nucleolin. Non-antisense antitumor effects of certain GROs such as inhibition of cell proliferation and induction of cell death have long been identified in various cell lines derived from solid tumors, leukemias and lymphomas 40. While the exact mechanism for these observations is still not fully elucidated 40, 148, 149, clinical trials with AS1411, which was initially developed as the first anticancer aptamer under the name AGR1000 by Aptamera commenced in 2003. A phase II study in patients with advanced renal cell carcinoma (RCC) that had not responded to tyrosine kinase inhibitor therapy, however, showed no significant anticancer effects 150. Despite this, AS1411 is now one of the most often used aptamers for imaging and targeted drug delivery, with less focus on the innate antiproliferative effect 151. Other therapeutic aptamers in the clinical pipeline include aptamers for macular degeneration, diabetes, inflammation, coagulation disorders, inflammation and oncology 152. Hence it is not surprising to see increasing efforts to combine the therapeutic effects with molecular imaging. In the following paragraphs, we highlight recent developments in this field.

A novel RCC binding DNA aptamer with potential theranostic use was developed by Zhang *et al.* 58. Interestingly, the aptamer was not selected by a classical SELEX protocol but derived from a library of 50 rationally designed nucleotide sequences that form unique stem and loop structures. SW-4, with a K_d_ of 45.92 ± 5.58 nM against 786-O cells was further truncated to improve tissue penetration and reduce cost for synthesis. The resulting SW-4b demonstrated improved binding with a K_d_ of 32.47 ± 2.3 nM and no off-target binding to embryonic kidney and normal tubular kidney cells (293T and HK-2, respectively). A competition assay with increasing amounts of SW-4 showed that SW-4b binds the same epitope, and proteinase treatment before incubation suggests that the target is likely an extracellular protein. The molecular target of SW-4b, however, is still unknown. The binding ability of Cy5-labeled SW-4b was tested by *in vivo* and *ex vivo* fluorescence imaging of mice bearing 786-O xenografts. Zhang *et al.* also investigated the physiological effects of SW-4b. The aptamer specifically inhibited cell proliferation of 786-O cells with an IC_50_ of 4.7 µM over a period of 72 h while no inhibition was observed for ssDNA control sequences or in control cell lines. Flow cytometry of 786-O cells treated with 10 µM library ssDNA or SW-4b revealed that more cells remained in the S-phase (26.29 ± 1.48 vs. 38.89 ± 0.19%). Even though these results are preliminary and the used concentration is arguably rather high, it is encouraging to see aptamers with the potential of being used for more than simple target recognition 5.

CLN0003 is a DNA aptamer that was selected against Jurkat cells *via* cell-SELEX and is targeting C-met, which interacts with hepatocellular growth factor and is believed to play a role in tumor metastasis 153. SL-1, representing the minimal binding motif against C-met was derived from CLN0003 in 2014 154. In addition to a K_d_ of 123 ± 26 nM against C-met-positive SNU-5 cells, SL-1 inhibits migration of SUIT-2 cells. To further explore the suitability of SL-1 as a theranostic agent, Zhang *et al.* functionalized the aptamer with Cy5 for the first account of multiple myeloma imaging in ARP-1 xenograft mice 155. Strong tumor uptake was visible from 10 to 60 min p.i., followed by a gradual decrease over the next 2 h. The therapeutic effect of SL-1 was investigated more thoroughly *in vitro*. In a co-culture model with human bone marrow HS5 cells, SL-1 inhibited cell proliferation of multiple myeloma cells in a dose dependent fashion and reduced cell migration and c-met signaling. In combination with bortezomib, a first-line drug against MM, SL-1 showed synergistic effects against multiple myeloma cells.

Yonping Jiang *et al.* reported the discovery of the *in vivo* selected RNA aptamer RA16 that specifically targets and binds human non-small cell lung cancer (NSCLC) cells and is able to inhibit cell growth *in vitro* and *in vivo* 9. For selection purposes a library of PEGylated RNA-oligomers containing 2'-fluoropyrimidines was injected into mice bearing NCL-H460 xenografts and reverse transcribed into DNA after extraction of tumors. RA16 was found to be highly enriched after 11 rounds of selection and can readily be generated by* in vitro* transcription. Fluorescently labeled RA16 bound NCL-H460 cells with a K_d_ of 9 ± 2 nM and was able to accumulate in subcutaneous tumors as shown by fluorescent imaging. In addition, RA16 inhibited tumor growth by 54.26%. Furthermore, an epirubicin adduct of PEGylated RA16 lead to a tumor growth inhibition of 64.38%. The aptamer was further investigated in a follow up study to compare chemically synthesized RA16 with *in vitro* transcribed RA16 and a truncated version of the sequence 59. Interestingly the K_d_ of synthetic and transcribed RA16 against NCL-H460 cells was found to be 24.75 ± 2.28 nM and 12.14 ± 1.46 nM, respectively, although it should be mentioned that the synthetic version was fluorescently labeled *via* streptavidin-PE as opposed to direct FITC labeling in the previous paper. Fluorescent imaging of both versions showed identical results, namely uptake in tumors from 0.5 to 3.5 h p.i. and high background fluorescence in excreting organs. Quantification of RA16 levels after organ extraction showed 50- to 1000-fold increased recovery for tumors compared to lung, heart, liver and kidney. Although the exact molecular target of RA16 remains to be elucidated, *in vitro* endocytosis studies of RA16 suggest internalization of the aptamer. Taken together, the two studies suggest a potential therapeutic and diagnostic application of RA16. More importantly, the strong difference between subjective image quality and the excellent tumor uptake rate by PCR from extracted organs highlights the difficulty of *in vivo* imaging with fluorescent aptamers.

A novel CD133 targeting aptamer for treatment of anaplastic thyroid cancer, termed AP-1-M, was selected using cell-SELEX against CD133 expressing Hek293 cells, with counterselection against non-CD133 transfected Hek293 cells 60. The aptamer had a K_d_ of 101.4 nM against CD133 expressing FRO anaplastic thyroid cancer cells. FRO xenograft bearing mice showed tumor uptake of Cy5.5-labeled AP-1-M up to 48 h, while no tumor uptake was observed for non-CD133 binding S30 aptamer controls. For cancer treatment, AP-1-M was loaded with DOX and injected into FRO xenografts every other day for 2 weeks. While unloaded AP‑1‑M showed a tumor inhibition rate of only 17.7%, the tumor inhibition rate of DOX-loaded AP-1-M and free DOX with 57.8 and 57.4%, respectively, suggests no improved treatment efficacy, although cell toxicity was reduced *in vitro* compared to free DOX.

**Figure 6:**
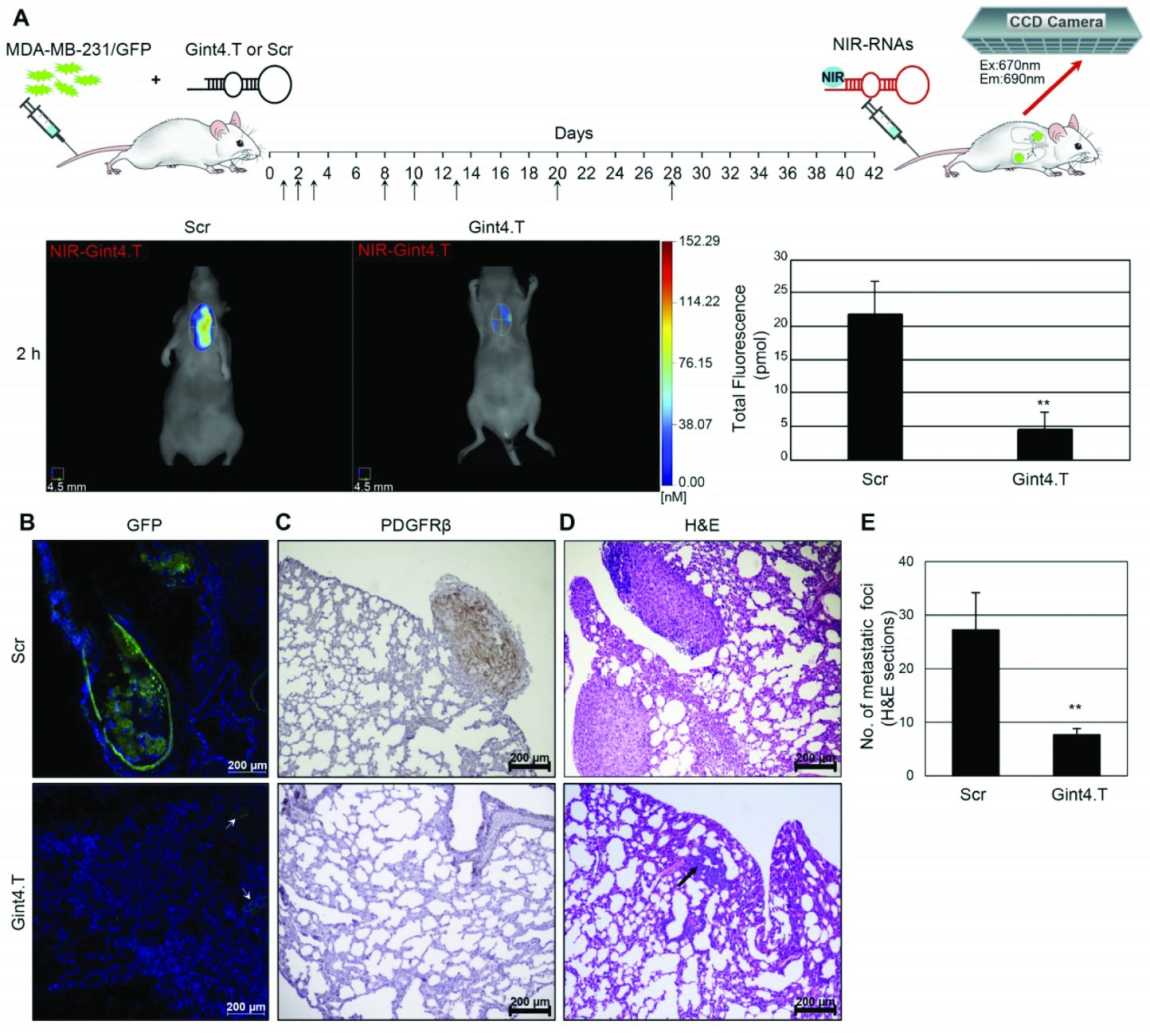
Gint4.T inhibits lung metastasis. (A) Mice were injected with MDA-MB-231 cells that were pre-incubated with Gint4.T for 30 minutes to establish a lung metastatic tumor model. On days indicated by an arrow, mice were i.v. injected with 1.4 nmol Gint4.T or scrambled sequence (Scr). On day 42 mice were injected with 1 nmol NIR-Gint4.T or NIR-Scrambled and analyzed with in vivo fluorescence imaging. (B) Lung sections of mice treated with Gint4.T or Scr, shown is the GFP channel from the MDA-MB-231 cells. (C) PDGFRβ immunostaining, (D) H&E staining, (E) number of metastatic foci in Gint4.T or Scr treated animals. Adapted with permission from 158. Copyright 2018 Ivyspring.

Originally developed by Laura Cerchia *et al.* in 2014, Gint4.T is an RNA aptamer that binds to the extracellular domain of platelet-derived growth factor receptor β (PDGFRβ) with antiproliferative effects on glioblastoma and the potential as a targeting vector for NP-based drug delivery 156, 157. Expanding the range of indications, they further assessed Gint4.T as a theranostic agent for molecular imaging and inhibition of metastasis in TNBC 158. Gint4.T effectively inhibited growth of PDGFRβ-positive MDA-MB-231 and BT-549 cells in 3D cultures and suppressed mesenchymal cell migration and invasion in the aforementioned cells in a transwell migration assay, while the invasiveness of PDGFRβ-negative BT-474 cells remained unchanged. *In vivo* molecular imaging of VivoTag-S 680 NIR-dye labeled Gint4.T in MDA-MB-231 xenografts showed strong tumor uptake 15 min and 2 h p.i. and remains detectable for up to 4 h. Importantly, background fluorescence was extremely low, highlighting the benefit of NIR dyes compared to standard fluorophores. Binding specificity was further confirmed by a complete lack of tumor fluorescence in a blocking study, in which animals received an excess of unlabeled Gint4.T prior to the NIR-labeled oligonucleotide. In a metastatic TNBC model, mice were injected with GFP expressing MDA-MB-231 cells *via* the tail vein followed by repeated administration of Gint.4T. Fourteen days after the last treatment, mice imaged with NIR-labeled Gint4.T showed an approximately 4-fold decreased fluorescence in the lung compared to mice treated with scrambled sequence. Immunohistochemistry and fluorescence microscopy confirmed reduced tumor growth and number of metastatic foci (Figure 6). This well-designed study highlights the potential of Gint4.T as a true theranostic agent and gives grounds for optimism that aptamers may indeed take on roles as “chemical antibodies”.

### 5.2 Drug Delivery with Aptamer Targeted Nanomaterials and Conjugates

In contrast to intrinsically therapeutic aptamers mentioned above, aptamers are often used as targeting agents for nanoformulations such as lipid nanoparticles, SPIONs, polymers or metal NPs. Many of these formulations can be further loaded with drugs and reporter molecules for the purpose of targeted drug delivery or imaging. Generally, the aim of actively targeted nanoformulations is to specifically bind target cells via overexpressed molecular target molecules. This concept of targeted nanomedicines has been explored for a variety of targeting agents such as antibodies, proteins, peptides, small molecules and it is not surprising to see an increasing use of aptamers for this purpose. In fact aptamers and nanomedicines look back at a long history of mutual development 159. In this section we summarize recent developments of aptamer targeted nanomaterials for theranostics.

Many cytotoxic drugs are characterized by poor pharmacokinetics and high toxicity, limiting their therapeutic use. Formulation strategies like encapsulation in nanoparticles is an effective strategy to reduce dose-limiting toxicity and several formulations have entered the market or are being evaluated in clinical trials 160. Employing a similar strategy to reduce side effects and improving the poor bioavailability of cisplatin, Agnello *et al.* co-encapsulated cisplatin and Cy7 into polymeric nanoparticles. To confer specificity, the NP formulation was targeted to EGFR *via* CL4 aptamer. Mice with subcutaneous MDA-MB-231 xenografts showed remarkably high tumor fluorescence 30 and 60 min p.i., compared to non-functionalized or scrambled ssDNA-functionalized NPs. Over the course of 24 h, the tumor signal decreased but remained significantly higher than the non-targeted formulations. Improved tumor targeting was further confirmed by strong growth inhibition of the targeted formulation compared to free cisplatin or non-targeted NPs.

Akbarzadeh *et al.* developed EpCAM aptamer EP1 161 targeted mesoporous silica coated QDs for DOX delivery and imaging of breast cancer 162. Compared to free DOX, cells were better protected from toxic effects using the aptamer targeted drug delivery system. Furthermore, the nanoparticle formulation showed significantly higher tumor growth inhibition compared to non-targeted particles or free DOX suggesting a beneficial effect of EpCAM aptamer mediated targeting. However, T_1_ weighted MRI remained qualitative since no image analysis was performed and a single time point of 24 h post injection was reported. While fluorescence imaging was only performed *ex vivo*, the results indicate more than 2-fold increased signal intensity of targeted QDs in the tumor region of 4T1 tumor bearing mice 24 h p.i., compared to non-targeted QDs. The same group published another paper in which the aforementioned QDs were encapsulated in DOX loaded polyethylene glycol-polycaprolactone PEG-PCL polymersomes and targeted with AS1411 163. While increased cellular toxicity of aptamer targeted polymersomes was observed in 4T1 and MCF-7 cells, some protection from non-specific free DOX toxicity was also seen. As in their previous study, imaging by MR fluorescence in 4T1 tumor bearing mice showed distinct contrast enhancement in the tumor both after 12 and 24 h, although no quantification of the signal intensity was attempted. In direct comparison, this formulation resulted in even stronger growth inhibition of subcutaneous xenografts than the previous formulation.

An albumin based theranostic agent, used BSA NPs co-loaded with DOX and indocyanine green (ICG) and decorated with AS1411 aptamer and KALA cell penetrating peptide for targeted multimodal cancer therapy and imaging 164. While stringent controls were missing, the data suggests optimal tumor to background ratio of the formulation at 24 h post injection. Complete tumor growth inhibition and even shrinkage was observed after a single administration of the formulation followed by photothermal therapy.

A study by Moghadam *et al*. used AS1411 targeted SPIONs coated with the iron chelator deferasirox for cancer treatment and MRI 165. The formulation in C26 xenografts moderately suppressed tumor growth compared to untargeted SPIONs and free deferasirox. MRI quantification was missing and an overall poor image quality did not allow for an evaluation of the nanoprobe as an imaging agent.

Antibody drug conjugates are an established approach to reduce off-target toxicity of highly potent drugs by specifically targeting the drug to the location of the disease. The same can principally achieved using aptamers. An example of such an aptamer-drug conjugate was reported by He *et al.* who used an AS1411-triptolide conjugate for targeted therapy of TNBC 166. Triptolide is a promising cytotoxic drug, although its unfavorable physicochemical properties and high toxicity have prohibited broad applications thus far. Specific tumor uptake of the Cy5-labeled conjugate was confirmed in MDA-MB-231 xenografts up to 8 h. Strong tumor growth inhibition without obvious side effects was observed, raising the prospect of other aptamer drug conjugates to become competitive to the extraordinarily successful antibody drug conjugates in recent years 167.

A theranostic SPECT probe for the treatment of PSMA-positive prostate cancer was reported by Jiao *et al*. 168 who hybridized the PSMA specific RNA aptamer A10-3.2 to MDM2 siRNA and functionalized it with the chelator SHNH for labeling with ^99m^Tc 168. The RNA chimeras were labeled with high radiochemical yield of 61.47% and a purity of >95%. The diagnostic potential was evaluated by SPECT in mice bearing PSMA-positive 22Rv1 or PSMA-negative PC-3 xenografts, showing a tumor to muscle ratio of 2.47, 3.42 and 4.63 and 1.31, 1.39 and 1.32, respectively after 0.5, 1 and 2 h. As a control, [^99m^Tc]TcO_4_^-^ was injected in both tumor models and showed similar tumor to muscle ratios as the chimera in PC-3 tumors. While both animal models showed high liver and kidney uptake, a significantly higher tumor uptake of 6.9, 8.44 and 9.71% ID/g in 22Rv1 xenografts, compared to 3.5, 2.68 and 1.91% ID/g in PC-3 xenografts, was observed in a biodistribution study. The therapeutic evaluation in 22Rv1 xenografts treated daily with the chimera showed a dose-dependent inhibition of tumor growth with no apparent side effects.

A new class of theranostic agents, termed aptamer-PROTAC conjugate (APC) was described by He *et al*. 169. APCs are prodrugs composed of proteolysis targeting chimeras (PROTACs), functionalized with a targeting aptamer through a GSH-reducible linker. PROTACs are bifunctional compounds that promote degradation of a target protein *via* binding to E3 ligase, which tags the protein of interest with ubiquitin for subsequent ubiquitin-proteasome mediated degradation. The proof-of-concept study used PROTACs specific for BET family proteins, which are related to gene transcription. Upon AS1411 mediated cell internalization, GSH reduces the cleavable linker to allow the PROTAC to bind its target protein and E3 ligase which activates the degradation cascade. Cy3-labeled APCs showed strong tumor uptake up to 8 h, while no uptake was observed for the same APC with control ssDNA in MCF-7 xenografts. Compared to free PROTAC, the APC resulted in better tumor growth inhibition, suggesting the potential use of APCs as novel therapeutic agents.

Doxorubicin and other related drugs are able to non-covalently intercalate into double stranded portions of aptamers 160, 170, 171. Some aptamers thus allow for facile loading of intercalating anti-cancer drugs for targeted drug delivery and theranostic applications. Nosrati *et al*. used AS1411 targeted SPIONs and loaded the aptamer with DOX for MRI and therapy of colon carcinoma 172. While *in vivo* MRI in C26 xenografts could not clearly separate AS1411 and scrambled ssDNA conjugated SPIONs, the targeted formulation showed a significantly better tumor growth inhibition. The difference in therapeutic efficacy and subjective imaging result of this study highlights the importance of adequate image analysis.

Since their discovery, many authors have drawn comparisons between aptamers and antibodies, emphasizing the facile synthesis and chemical stability of aptamers combined with high binding affinity and specificity. This has led to aptamers often being referred to as “chemical antibodies”. A study by Yong Serk Park *et al.* directly compared the tumor targeting ability of theranostic lipid NPs co-encapsulating quantum dots and paclitaxel, using either the anti EGFR antibody cetuximab or an undisclosed anti EGFR aptamer for targeting (immuno-QDM or aptamo-QDM, respectively) 173. While tumor fluorescence stayed constant for immuno-QDM and aptamo-QDM treated mice, a rapid decrease in intensity was observed for untargeted QDMs. Notably, tumor to liver ratio of immuno-QDMs and aptamo-QDMs was >1 at 48 h p.i. and reached a value of 2.18 and 2.18 after 72 h, respectively, while the tumor to liver ratio for non-targeted QDMs decreased to 0.43 over the same time frame. In terms of therapeutic efficacy, both aptamo-PTX-QDMs and immuno-PTX-QDMs were equally effective in tumor growth inhibition and slowed tumor growth approximately twice as effective as free paclitaxel or untargeted NPs. This theranostic study showed almost identical pharmacokinetic profiles for aptamo-QDMs and immuno-QDMs, corroborating the often-repeated claim that aptamers could replace antibodies. Building upon the same targeting system but using co-encapsulated siRNA instead of paclitaxel, the same team of researchers prepared lipid NPs co-encapsulating quantum dots and siRNA (QLs) for the treatment of TNBC 174. With a size of approximately 175 nm for the different formulations (immuno-QLs, aptamo-QLs and untargeted NPs) the formulations were considerably larger than the approximately 40 nm sized formulations in the previous study. Interestingly, the pharmacokinetic distribution of the formulations in MDA-MB-231 xenografts differed considerably from the previous formulation with notable lung uptake, potentially due to initial agglomeration in the lungs. Background fluorescence remained high throughout the duration of the fluorescence imaging study and tumors were less clearly delineated than in the previous study. Moreover, tumor to liver ratios, albeit statistically higher for both targeted formulations compared to untargeted NPs, failed to reach a value above 1 throughout the course of the study. Interestingly, immuno-QLs showed marginally better tumor to liver ratios than aptamo-QLs. In accordance with the moderate influence of targeting on tumor imaging, the therapeutic effect of aptamo- and immuno QLs loaded with siRNA was comparable to that of untargeted NPs. Since size of the lipid NPs and the tumor model are the most obvious difference in these studies, it can be concluded that although aptamer targeted NPs can theoretically be employed for targeted delivery of NPs, a multitude of factors must be considered for the development of imaging and therapeutic probes.

### 5.3 Aptamer Targeted Nanomaterials for Therapy and Molecular Imaging

While some nanoparticles serve as carriers for drugs and other payloads, the inherent physicochemical properties of certain materials allow for their use as therapeutic agents directly. Noteworthy in this regard are superparamagnetic NPs that can be used for MRI or for magnetodynamic therapy (MDT) by applying an alternating magnetic field (AMF) to inductively heat the superparamagnetic NPs and thermally ablate cancer cells. Kolovskaya *et al.* synthesized arabinogalactan stabilized superparamagnetic NPs for MRI and treatment of cancer 175. Tumor specific targeting was achieved through conjugation of AS-14 andAS-42 aptamers, which bind to fibronectin and Hsc70, respectively 176, 177. To evaluate the imaging properties of the nanoprobe, subcutaneous and intracranial Ehrlich ascites carcinomas were established in mice. Qualitatively, similar contrast enhancement of the nanoprobe and Omniscan^TM^ could be detected 30 min after intravenous injection, although the formulation seemed to better visualize the brain tumor, suggesting its potential use as a brain tumor imaging agent. Assessed by histology, the aptamer-targeted group also showed strong tumor necrosis after MDT.

Another formulation for MDT therapy by Chen *et al.* used AS1411 targeted zinc-doped iron oxide nano octahedra co-loaded with DOX and HSP70/HSP90 siRNA 178. Remarkable tumor growth inhibition in 4T1 xenografts was achieved after a single injection of the nanoprobe and AMF application. Fluorescence imaging showed peak tumor uptake after 8 h. While the signal remained nearly constant up to 48 h in the AS1411-functionalized probe, a rapid decrease was observed in the non-targeted group, highlighting the benefit of AS1411 mediated active targeting. T_2-_weighted MRI initially showed comparable tumor to muscle ratios between both groups, but significantly increased ratios for the targeted probe at 24 and 48 h, corroborating the results of the fluorescence imaging.

PTT uses electromagnetically induced heating to kill cancer cells. Metal nanomaterials such as gold nanoparticles are able to produce heat under specific wavelengths which allows precise spatiotemporal control of PTT. Along with the prolific work of purely diagnostic MRI contrast agents discussed in section 3.1, Wang *et al*. synthesized a theranostic probe for dual fluorescence and MRI with the capacity for PTT 179. Briefly, the AS1411 targeted nanoprobe consists of gold nanobipyramids, decorated with gold nanoclusters and Gd_2_O_3_ for fluorescence imaging and MRI, respectively. While the inherent fluorescence of the gold nanoclusters was only employed for *in vitro* imaging, MDA-MB-231 xenograft bearing mice showed markedly increased T_1_ signal intensity peaking at 4 h. A similar profile but overall lower contrast enhancement was observed for non-targeted NPs, suggesting a beneficial effect of active targeting compared to EPR mediated uptake. PTT showed excellent tumor growth inhibition but negligible difference in targeted vs. non-targeted groups.

Sonodynamic therapy uses US waves for the generation of ROS. An example of a theranostic platform for sonodynamic therapy using AS1411 for targeting was reported by Wang *et al.* who loaded hematoporphyrin monomethyl ether (HMME) and acriflavine into liposomes that were surface coated with MnO_2_ nanosheets 180. HMME serves as sonosensitizer and generates ROS upon US irradiation, while hypoxia induced upregulation of VEGF and cancer cell proliferation was prevented by acriflavine, an inhibitor of hypoxia inducible factor-1α. The nanoprobe demonstrated effective cell uptake and cancer cell toxicity in combination with US irradiation in SKOV-3 cells. Flow cytometry revealed 30.6% apoptotic cells after treatment, however, no control cells were included to compare efficacy in normal cells, which is especially concerning since cell viability seems to be compromised also in cells treated with untargeted nanoprobes. Sonodynamic therapy showed almost complete growth inhibition in mice undergoing US irradiation 12 h post injection of the formulation. Mn^2+^ is a strong T_1_ shortening contrast agent and based on the assumption that the MnO_2_ nanosheets are reduced in the TME, the authors hypothesized the nanoprobe could be used for targeted MRI contrast enhancement. MRI images showed time dependent contrast enhancement of subcutaneous SKOV-3 tumors up to 12 h. However, no further quantification was performed and high background signal in liver and intestinal system was observed for targeted and untargeted liposomes.

Another probe for PTT and contrast enhanced US and photoacoustic imaging used AS1411 targeted, PLGA-encapsulated liquid fluorocarbon perfluoropentane and iron(II) phthalocyanine (FePC) 181. FePC promotes transition of the liquid perfluoropentane core to the gas phase which enables contrast enhanced US imaging. *In vivo* photoacoustic imaging in MCF-7 xenografts peaked in the tumor after 6 h, while EPR mediated accumulation of untargeted NPs exceeded the preinjection signal only marginally after 24 h. Contrast enhanced US was evaluated 6 h post injection of the NPs following NIR irradiation of the tumor region to promote the liquid to gas phase transition of the particles. Targeted particles showed significantly increased contrast upon irradiation, while no difference was observed for non-targeted particles. PTT resulted in complete tumor remission in mice injected with targeted NPs while a strong but slightly less pronounced growth inhibition was observed with non-targeted NPs.

A targeted radiosensitizer used AS1411 modified PEGylated silver nanoparticles for radiation therapy of glioblastoma 182. A Cy5 labeled version of this construct was injected into mice bearing intracranially implanted C6 glioblastomas to evaluate the tumor targeting ability of the radiosensitizer *in vivo*. The accumulation of AS1411-targeted NPs in tumors was superior to untargeted NPs and remained detectable for 48 h with maximum brain uptake at 6 h. Throughout the course of the study, the background fluorescence remained high and was still detectable in liver and kidneys after 48 h. *Ex vivo* imaging confirmed localized tumor uptake of aptamer-targeted NPs after 48 h, while no fluorescence was detectable for untargeted NPs. Importantly, the median survival time of glioblastoma bearing mice receiving AS1411-targeted silver NPs in combination with 6 Gy of radiation 6 h post injection was significantly prolonged to 45 days, compared to 24 days for mice receiving only radiation treatment.

### 5.4 Aptamer Functionalized Smart Nanomaterials

Similar to the aforementioned “smart” imaging probes, some theranostic formulations use more sophisticated approaches to reduce off-target toxicity through stimulus responsive activation mechanisms. In the following paragraph recent developments of such probes are briefly discussed.

**Figure 7:**
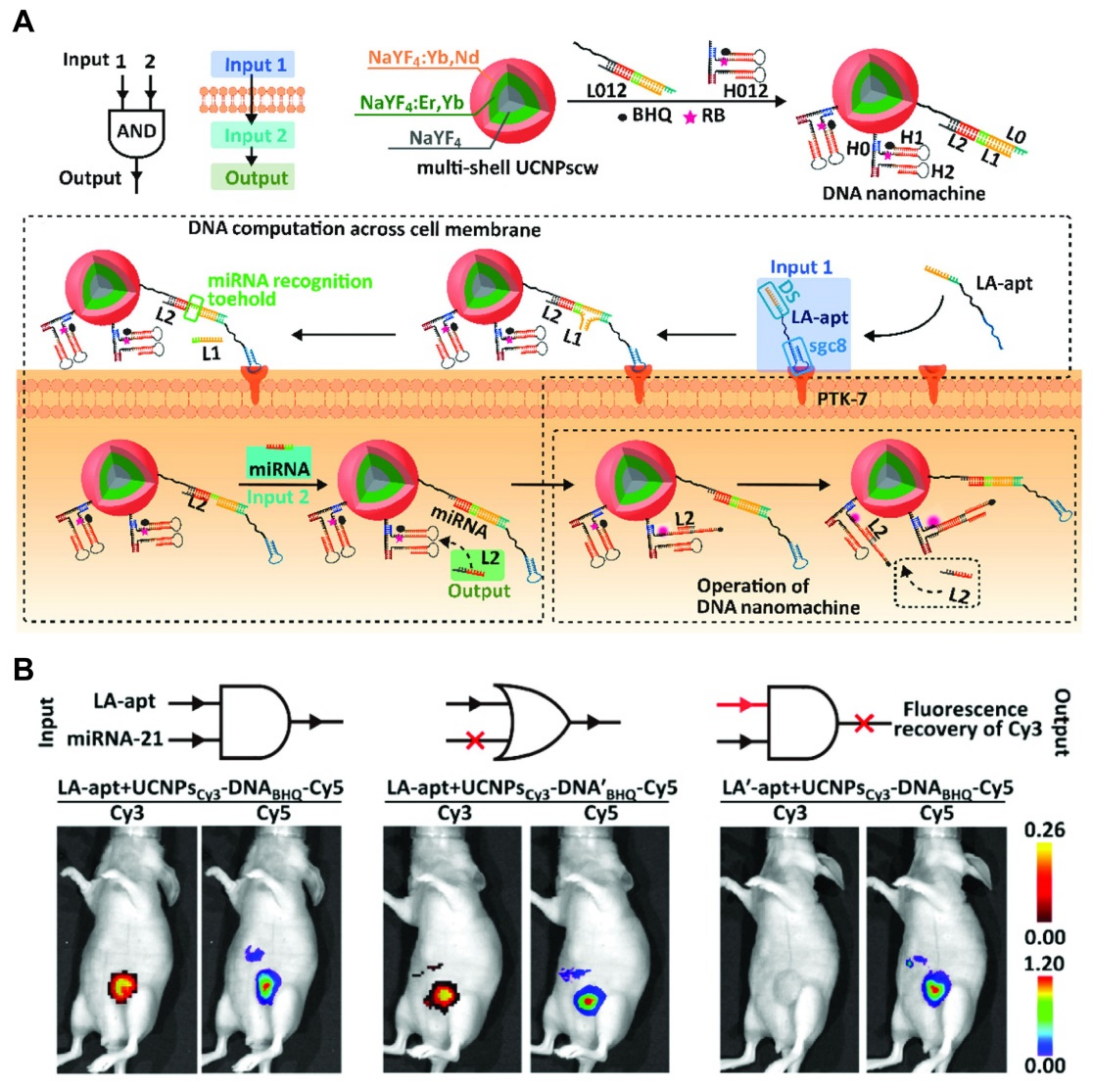
DNA logic gate for tightly controlled activation of fluorescence and ROS generation. (A) Construction and mechanism of activation of DNA nanomachine: Binding of modified sgc8 (LA-apt) constitutes input 1. UCNPs hybridize with receptor bound LA-apt for internalization. The second input is binding of endogenous miRNA which releases the L2 strand. L2 can hybridize with H012 to remove the quenching effect of BHQ on the photosensitizer Rose Bengal (RB). For imaging RB is replaced with Cy3 to monitor fluorescence activation in vivo. (B) In vivo imaging in mice bearing dimethylbenzanthracene-induced breast cancer 4 h post injection of the nanomachine. UCPNs were labeled with Cy5 to monitor tumor uptake. Injection of LA-apt + UCNPs_Cy3_-DNA_BHQ_-Cy5 shows activation of Cy3 fluorescence limited to the tumor. LA-apt + UCNPs_Cy3_-DNA'_BHQ_-Cy5, a version that does not require input 1 for activation is activated also outside of the tumor, indicated by fluorescence in the liver. LA'-apt, with additional miRNA blocking region serves as a control by preventing miRNA mediated activation while retaining tumor targeting. Adapted with permission from 183. Copyright 2021 American Chemical Society.

Zhang *et al*. achieved exceptionally low background fluorescence by utilizing upconversion luminescence NPs (UCNPs) with a built in logic gate that necessitates two separate inputs for fluorescence imaging and PDT of breast cancer tumors 183. Conceptually, the probe works in a two-step mechanism that requires binding of modified sgc8 aptamer to cancer cells, followed by the injection of multishell UCNPs which hybridize with the modified sgc8 aptamer. Once the probe is internalized, miRNA-21 serves as a second input signal for the release of an inhibitory element for fluorescence activation and ROS generation (Figure 7). The complexity of the nanomachine as well as the need for two separate injections represent a considerable obstacle for possible clinical translation. Nevertheless, the *in vivo* results show impressively specific tumor imaging with low background and strong tumor growth inhibition in mice bearing dimethylbenzanthracene-induced breast cancer.

Based on their SAAP approach from 2016 184, Lei *et al.* developed a DNA nanotriangle delivery system for fluorescence imaging and drug delivery. Briefly, the probe consists of fluorescently labeled sgc8c aptamer hybridized to a complementary strand containing a quencher. Upon binding to PTK7 and subsequent conformational change of sgc8, the inhibitory strand dissociates from the labeled aptamer strand and the fluorescence signal is activated. In the present study the above mentioned activatable probe is linked to a self-assembling DNA-nanotriangle to increase avidity and to allow intercalation of DOX in the nanotriangle backbone as payload 185. This theranostic probe was assessed in an *in vivo* fluorescence imaging study including excellent controls in mice bearing CCRF-CEM (PTK7 positive) or Ramos (PTK7 negative) tumors. Upon injection of the nanoprobe (NTri-SAAP*), tumor uptake in CCRF-CEM mice was observable already after 10 min, and the fluorescence signal peaked after 60 min. Over the course of the study, a gradual increase of background fluorescence due to the degradation and elimination of the probe, predominantly through liver and to a lesser extent kidneys, was observable. In comparison, the split activatable probe without DNA-nanotriangle carrier showed a similar tumor uptake but substantially higher background fluorescence and an overall faster elimination. NTri-SAAP* injected into non-tumor bearing mice or Ramos tumor bearing mice, as well as CCRF-CEM tumor bearing mice injected with a probe that was functionalized with a control sequence did not show any tumor uptake. Although the imaging study remains qualitative it clearly shows an improved tumor to background ratio for the nanotriangle and an altered elimination profile through hepatic elimination compared to the free SAAP. The anti-tumor effect of the nanoprobe was assessed in mice bearing CCRF-CEM tumors administered 2 mg/kg free Dox or NTri-SAAP*-Dox every two days for 13 days. Compared to control mice injected with PBS or Ntri-SAAP*, tumor growth was strongly inhibited in both DOX treatment arms and a small but statistically significant better tumor suppression was observed in the NTri-SAAP*-Dox group. This well-designed study is highlighting the potential of “smart” delivery systems for imaging and therapy to mitigate the unfavorable pharmacokinetic profile of unmodified aptamers due to their rapid elimination and chemical instability.

Exploiting a unique property of the TME, namely hypoxia and high hydrogen peroxide levels, Weihong Tang *et al.* sought to develop a theranostic probe for fluorescence imaging and PDT consisting of AS1411 with intercalated hemin moieties, which catalyzes the production of O_2_ from H_2_O and 5'-pyrochlorophyll A (PA)-modification for the generation of ^1^O_2_ under NIR light irradiation 186. The therapeutic potential was examined in mice bearing subcutaneous MCF-7 tumors, receiving PDT 5 h after injection of the probe on day 0 and day 6. Compared to saline, a substantial retardation of tumor growth and a roughly 3-fold reduction of tumor weight was recorded at day 16. The fluorescent properties of PA allow for *in vivo* imaging, which revealed high liver and kidney uptake of both free PA and the nanoprobe while tumor uptake was only observable for the aptamer probe. A biodistribution study of organs 5 h p.i. showed tumor uptake of 5.2% ID/g which is higher than most organs but considerably lower than liver and spleen (approximately 30% and 12% ID/g, respectively).

Similarly utilizing the non-physiological properties of the TME, Wu *et al.* devised a staggered tumor targeting approach based on EPR mediated uptake of hyaluronic acid encapsulated AS1411 aptamer loaded with DOX and subsequent liberation of the payload *via* cleavage of the shell due to the high concentration of hyaluronidases in the TME 187. Due to the fluorescent properties of DOX, imaging can be performed in mice treated with free DOX, as well as free or hyaluronic acid encapsulated DOX loaded AS1411. Despite good tumor growth inhibition and tolerability of hyaluronic acid encapsulated AS1411-DOX *in vivo*, fluorescence imaging of mice bearing subcutaneous 4T1 tumors did not show clear tumor uptake at any time point (2, 4 or 8 h) due to high background fluorescence. Moreover, despite a tentatively higher DOX signal for hyaluronic acid encapsulated AS1411-DOX compared to free DOX or non-encapsulated AS1411-DOX in tumors harvested after the last imaging time point, no definitive conclusion can be drawn due to a lack of quantitative analysis.

Xin *et al*. synthesized a peroxide degradable nanocarrier encapsulating the synergistically acting compounds β-lapachone (LAP) and tirapazamine (TPZ) for TME activated therapy 188. Targeting was achieved through liver cancer specific TLS11a aptamer, which was demonstrated using ICG-labeled NPs in mice bearing subcutaneous HepG2 xenografts. Compared to untargeted NPs, the TLS11a targeted aptamers showed stronger and more persistent tumor uptake up to 48 h. The formulation showed strong tumor growth inhibition, although not much difference was observed between targeted and non-targeted groups.

Sun *et al*. reported a multimodal nanoprobe that utilizes the TME for triple therapeutic effect through PDT, chemodynamic therapy and PTT with MRI and fluorescent imaging capabilities 189. The AS1411 targeted probe consisting of gold nanocluster and ICG encapsulated by MnO_2_, is able to produce O_2_ in the H_2_O_2_-rich TME and releases its content through GSH-mediated degradation. In turn, the fluorescent properties of the gold nanoclusters are restored and Mn^2+^ catalyzes the production of hydroxyl radicals and serves as an MRI contrast agent, while the NIR fluorescent dye ICG catalyzes the production of ROS. *In vivo*, the nanoprobe was able to accumulate in MCF-7 xenograft bearing mice demonstrated by MRI and fluorescent imaging. Marginally higher tumor uptake was observed for the AS1411 targeted probe. After NIR irradiation only a slight difference in tumor growth inhibition was observed between targeted and non-targeted groups. The turn-on fluorescent properties of the gold nanoclusters were demonstrated by an increase of fluorescence after 16 h, although the excitation wavelength of 488 nm is arguably not optimal for *in vivo* applications. Despite strong tumor growth inhibition, the imaging capabilities require further optimization.

## 6. ATP Binding Aptamers for Molecular Imaging and Theranostics

A conceptually different class of aptamer-based nanomaterials for imaging or therapy are stimulus responsive probes where a binding event triggers an effect such as fluorescence activation or drug release. Notable examples include ATP triggered release of DOX 190 or the fluorometric sensing of platelet derived growth factor 191 or Cl^-^ ions 192. Here we discuss a range of ATP sensing nanoconstructs for ATP-dependent imaging 143-146 or theranostic applications using PDT 193, 194. In contrast to the other aptamers or aptamer-nanoconjugates discussed in this review, the ability to bind ATP in these constructs is not used for targeting, but to allow for molecular imaging through fluorescence activation upon the binding event. A common feature of the here discussed probes is the ATP triggered conformational change of the aptamer sequence which leads to a signal activation allowing for fluorescence imaging. In this sense, these “ATP-sensors” are universal imaging probes for cancer, although some more sophisticated designs confer additional specificity due to tumor specific miRNA that is required as additional input 144, 194. A common limitation for probes is the requirement of cell internalization for ATP binding, which makes tumor specific targeting and internalization a major challenge that remains to be addressed. This is reflected in the fact that in four of the six studies the nanoconjugates were injected intratumorally 143, 145, 146, 194. In the following section we discuss recent reports of such ATP binding molecular imaging and theranostic probes in preclinical studies.

**Figure 8:**
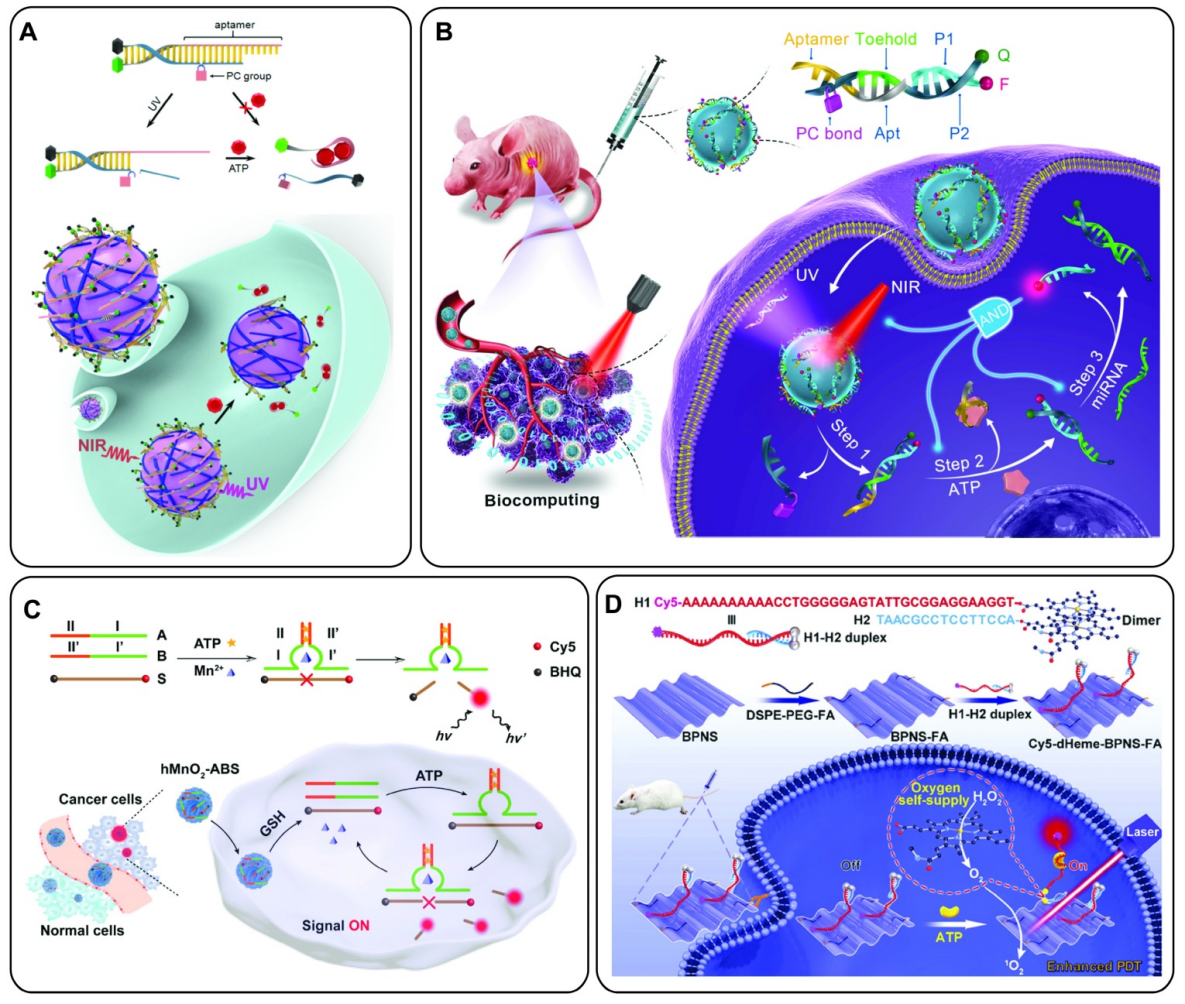
Schematic representation of ATP select ATP binding probes for molecular imaging and theranostics. (A) In the “off”-state, the Cy5-labeled ATP aptamer is hybridized to an inhibitory strand functionalized with a photocleavable group and a fluorescence quencher. Upon UV irradiation, the photocleavable group dissociates and allows binding of ATP and fluorescence activation. The double strand is electrostatically adsorbed to the surface of UCNPs which produce UV light to promote cleavage of the photocleavable linker inside tumor cells. Adapted with permission from 143. Copyright 2017 American Chemical Society. (B) The same UCNPs as in (A) are used to facilitate cleavage of the photocleavable linker (input 1). This allows binding of the ATP aptamer and dissociation from the complementary quencher strand (input 2). Tumor specific miRNA hybridizes with the quenching strand, releasing the fluorescently labeled P1 strand and activating fluorescence (input 3) for highly specific ATP intracellular ATP imaging. Adapted with permission from 144. Copyright 2021 American Chemical Society. (C) TME specific probe requiring the dual input of Mn^2+^ and ATP binding to restore the catalytic function of the split aptazyme I and I', cleaving the complementary S strand to activate fluorescence due to the dissociation of the quencher. Adapted with permission from 145. Copyright 2021 American Chemical Society. (D) Theranostic probe for enhanced PDT due to ^1^O_2_ generation. Tumor uptake is achieved via folate receptor mediated internalization. Intracellular ATP binding promotes structural switch of the H1 strand, “turning on” the fluorescence signal and promoting dissociation of the heme dimer into more active monomers to produce O_2_ from intracellular H_2_O_2_. The black phosphorous nanosheets catalyze 1O_2_ production for PDT. Adapted with permission from 193. Copyright 2018 Elsevier.

Focusing purely on molecular imaging, Lele Li *et al.* developed an NIR to UV upconversion luminescence (UCL)-activatable imaging probe for ATP sensing 143. Briefly, the imaging probe consists of an ATP binding Cy5-labeled aptamer that is hybridized to a complementary DNA strand containing a quencher and a photocleavable linker. The DNA is bound electrostatically to the surface of NaGdF4:70%Yb,1%Tm@NaGdF4 core-shell upconversion NPs (UCNPs). Upon NIR irradiation with 980 nm light, the thulium-doped UCNPs exhibit 320 and 360 nm UCL, which cleaves the photocleavable linker on the inhibitory DNA strand, thus facilitating dissociation of the strands upon ATP binding and subsequent increase of Cy5 fluorescence. Imaging of the UCNPs was performed in nude mice bearing subcutaneous HeLa xenografts. Thirty min after intratumoral injection of UCNPs, one group was irradiated with NIR light. The NIR irradiated group showed a 1.86 and 1.81-fold increased relative fluorescence at 2 and 4 h, respectively, whereas only a marginal increase was observed in the non-irradiated control group (Figure 8A). This innovative approach using a photoswitchable probe, rather than an “always on” approach, allows for excellent spatial control of fluorescence activation with the benefit of deep tissue penetration.

In an evolution of the previous design the same group devised a strategy that requires three separate inputs, namely NIR irradiation, ATP binding and tumor specific miRNA (miR21) binding to exhibit fluorescence activation (Figure 8B) 144. Conceptually, the nanodevice employs the same UCNP mediated cleavage of a photosensitive group as in the previous study. This allows for the binding of ATP by the aptamer, which in turn allows the tumor specific miRNA to bind to its complementary sequence functionalized with a fluorescence quencher. Only then, the Cy5-modified strand is released and able to emit fluorescence. Co-injection of the probe into the tumor and contralateral normal tissue of mice, NIR irradiation led to a rapid three-fold increased fluorescence signal of the tumor area while only a moderate increase in fluorescence was observed in the normal tissue. The same profile was observed in mice that received the same injections without NIR irradiation. Moreover, signal enhancement was realized after intravenous injection, while no increase was observed in the control groups lacking either one of the critical input signals, further showcasing the tightly regulated signal activation *in vivo*.

Shen *et al.* reported a theranostic nanoprobe that uses ATP and miRNA for fluorescence activation and ^1^O_2_ production for imaging and PDT 194. Mechanistically, the probe uses Y-motif DNA structures conjugated to the surface of folate functionalized CdTe/ZnS QDs (Y-motif/FA@HyNPs). Similar to a previous design 195, endogenous Let7a miRNA leads to a partial displacement of the Y-motif which allows binding of ATP and formation of a closed hairpin structure of the aptamer. This releases the miRNA and makes it available for subsequent activations. Furthermore, hairpin formation is accompanied by dissociation of an BHQ2-functionalized inhibitory strand from the Y-motif rendering the QDs fluorescent. This ATP fueled cascade activation leads to a marked increase in fluorescence and singlet oxygen production from the QDs at sub-nanomolar levels of Let-7a miRNA. *In vivo*, Y-motif/FA@HyNPs demonstrated both the capacity for intracellular Let-7a monitoring with fluorescence imaging as well as impressive antitumor effect after PDT. Upon intratumoral injection Y-motif/FA@HyNP reaches a maximum fluorescence intensity after 3 h which was maintained over 18 h, suggesting a gradual intracellular uptake and fluorescence activation. Interestingly, mice co-treated with a Let-7a miRNA inhibitor, exhibited a two-fold lower fluorescence signal. The antitumoral effect was evaluated in mice treated with Y-motif/FA@HyNPs and three cycles of PDT, leading to complete tumor growth inhibition, whereas almost no growth suppression was observed in mice co-treated with Let7a inhibitor.

Zhout *et al.* developed a nanoprobe that requires cooperative binding of Mn^2+^ and ATP to turn on fluorescence 145. Briefly, the probe uses a split DNAzyme and ATP aptamer which are adsorbed to MnO_2_ NPs. Upon binding of ATP, the two subunits self-assemble into the active conformation of the DNAzyme while the GSH-rich TME releases the cofactor Mn^2+^ from the NP carrier for catalytic activity. Finally, the DNAzyme cleaves a substrate strand that is functionalized with a fluorophore and quencher, thereby “turning on” the fluorescent probe (Figure 8C). Tumor fluorescence peaked5 h after intratumoral injection. More importantly, tumor fluorescence followed a similar profile even after intravenous injection and was detectable for up to 12 h.

Following a theranostic approach, Liu *et al.* developed an ATP-activatable nanoconjugate, composed of black phosphorus nanosheets (BPNS) for the production of singlet oxygen for PDT 193. Briefly, the construct is composed of a 5'-Cy5 and 3'-Fe-protoporphyrin IX (Heme) functionalized ATP aptamer hybridized to a partially complementary 5'-Heme functionalized strand, leading to the formation of a catalytically inactive Heme dimer. The double stranded construct is non-covalently adsorbed to the surface of BPNS modified with folic acid for tumor cell targeting and internalization. Upon binding of ATP, the DNA double strand dissociates, leading to an increase in fluorescence and generation of O_2_ from H_2_O_2_, catalyzed by the more active monomeric form of Heme. Finally, the BPNS produces ^1^O_2_ from the in situ generated O_2_ for enhanced PDT (Figure 8D). The fluorescence increase allows for monitoring of cellular internalization after intratumoral injection, suggesting a steady uptake of the formulation over a period of 48 h. PDT was evaluated in mice with bilateral tumor xenografts that received PDT in one tumor 24 h after the injection of the nanoformulation. Complete absence of fluorescence was observed in the irradiated tumor within 24 h of treatment and the tumor volume was reduced to 6% of the original size after 14 days.

In contrast to the previously mentioned probes that work through various forms of strand association or dissociation to promote fluorescence activation and other downstream effects, Chu *et al.* used a simpler approach that relies on conformational change of the ATP aptamer for fluorescence activation. ROX-labeled ATP aptamers were adsorbed to the surface of titanium carbide nanosheets with inherent fluorescence quenching properties. Upon ATP binding, the aptamer undergoes a conformational change, dissociates from the nanosheets and fluorescence is “turned on”. *In vitro*, fluorescence activation was observed in human derived plasma and mouse derived plasma and urine, spiked with low mM concentrations of ATP. Mice bearing 4T1 or MCF-7 showed significantly increased fluorescence compared to normal tissue sites 6 h after intratumoral or subcutaneous injection of the nanoprobe. High background fluorescence, however, indicates the need for fluorescent dyes with optimized spectral properties for *in vivo* imaging.

Compared to more traditional imaging probes, ATP-activatable nanoparticles are rather complex and it remains to be seen if similar probes for other imaging modalities than fluorescence can be developed. A notable improvement over directly labeled aptamers or nanomaterials, however, is the better specificity and tumor persistence which allows imaging with lower background and over longer periods than “always on” probes. Specifically, the incorporation of miRNA as co-activators offers the intriguing potential to create highly specific or personalized imaging or theranostic probes due to their aberrant expression profile in a variety of cancers 196. More research in the coming years will undoubtedly further substantiate the encouraging preliminary results of ATP binding aptamers for imaging and therapy.

## Conclusion

To date a small number of commercially available aptamer-based diagnostic tests in the agricultural industry and for oncology is available 152. Several diagnostic aptasensors for point-of-care testing are currently undergoing clinical trials and might result in widespread adaptation in clinical practice in the near future 198. For therapeutic use, pegaptanib remains the only aptamer that has reached FDA approval and entered the market to date 29. Despite its initial success following its commercialization in 2004, sales have been outpaced by the anti-VEGF antibodies ranibizumab (Lucentis) and off-label used bevacizumab (Avastin) due to better clinical response. It should be pointed out that during the time of its development, the role of different VEGF isoforms in ARMD was incompletely understood and pegaptanib was specifically selected to bind the VEGF_165_ isoform, whereas both antibodies additionally bind the VEGF_121_ isoform 199. Nevertheless, pegaptanib has unequivocally proven that aptamer therapeutics are able to compete on the market with antibodies. This is reflected in a large number of aptamer therapeutics in the therapeutic pipeline 152.

**Figure 9:**
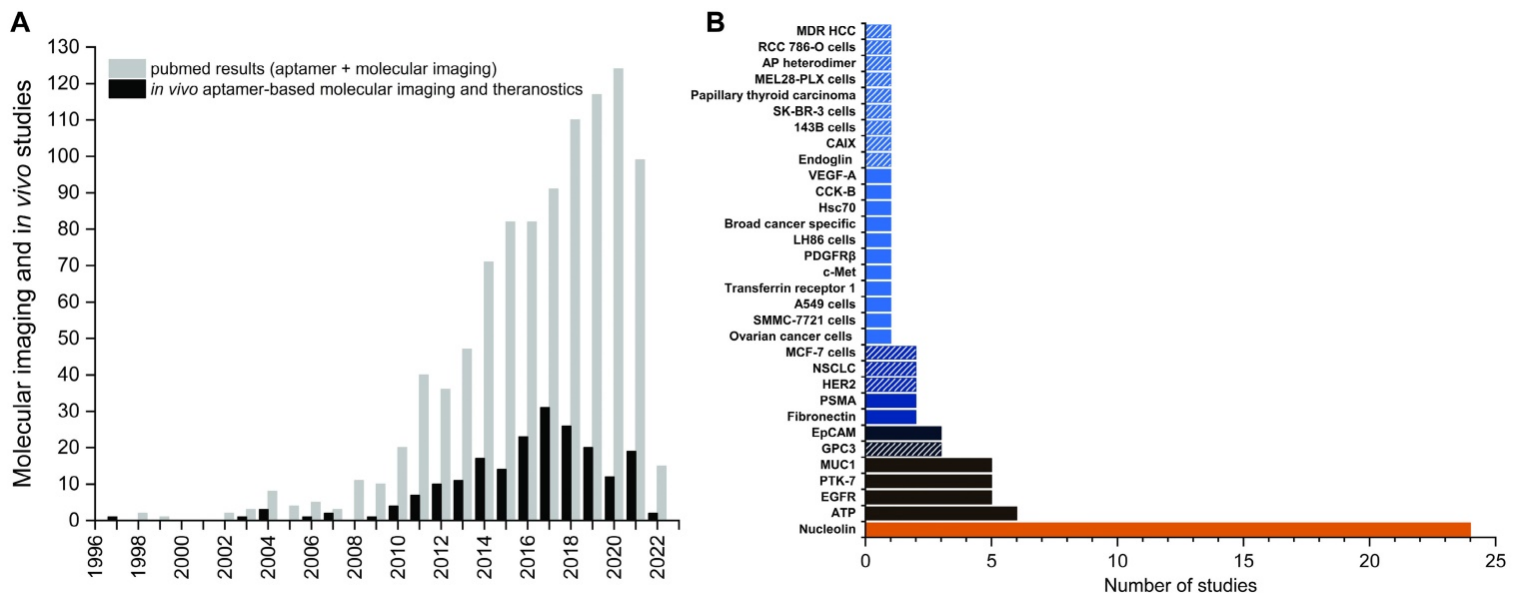
Aptamers for molecular imaging and theranostics in vivo. Due to the lack of human trials, “in vivo” refers to preclinical studies. (A) Pubmed search of aptamers in molecular imaging theranostics (grey bars) and individual studies with reported in vivo results in the last years (black bars). (B) Partition of molecular targets for aptamers in this review, novel aptamers shown in shaded bars.

With regards to molecular imaging, the preclinical examples discussed above and in previous reviews 35-37, 39, 200, as well as the output of scientific literature over the past 10 years indicate a strong interest to develop aptamer-based imaging probes (Figure 9A). Despite a steadily growing number of publications about aptamers for molecular imaging and theranostics, no aptamers for molecular imaging or theranostics have entered the market and a single clinical trial is currently evaluating ^68^Ga-labelled sgc8 in humans as a probe for colorectal cancer imaging 201. Considering the fact that the history of aptamers dates back 30 years, the slow clinical adaptation of aptamers as a whole and specifically for imaging applications is somewhat surprising.

There are several reasons for the slow development of aptamers. During the early days of aptamer development, intellectual property restraints undoubtedly took momentum out of the emerging technology and stalled progression before the research community fully explored the potential of aptamers 202. Another reason why aptamers are slow in entering routine clinical use is described as “thrombin problem” by Geoffrey Baird 203, which refers to the fact that a small number of “established” aptamers are being used instead of undertaking the tedious and risky task of selecting and adequately characterizing novel aptamers. This is reflected in the fact that more than half of all *in vivo* imaging studies reviewed by Bouvier-Müller and Ducongé in 2018 target the same four molecular targets, namely nucleolin, mucin 1, PTK7 and EGFR 39. The studies discussed in this review reveal a similar trend with a strong overrepresentation of the same targets (Figure 9B). Perhaps the biggest challenge that aptamers face, is that they are competing with antibodies, but unlike the latter cannot benefit from well-established manufacturing processes and years of experience and financial support within the industry. While these factors might partially explain why aptamers have been slow to reach the clinics, we don't think there are fundamental limitations of aptamer technology that prevent it from reaching its full potential.

Nevertheless, several key factors are crucial to further advance aptamers into clinical practice, including 1) the need for standardization in experimental design and imaging parameters, 2) the need for quantitative data from imaging studies and 3) the need for characterization of molecular targets of aptamers.

1) A general limitation in the evaluation of aptamers as imaging probes is that there seems to be a lack of standardization of imaging parameters and experimental design. Most importantly, the interval between injection and imaging, as well as the duration of the imaging can vary considerably between studies. While in some cases a single timepoint is shown, others show dynamic imaging studies over longer times. Especially for fluorescent probes it is common to see *ex vivo* images of selected organs, although the timepoint of organ harvest can again differ significantly between studies. Control experiments are also highly variable. Especially nanoparticle formulations should ideally include a control with a scrambled sequence of equal length to the aptamer to account for the EPR effect or other passive distribution and accumulation effects of the formulation. This is problematic since the physicochemical properties like size, shape, charge and other related parameters of the nanomaterial itself have a large influence on the pharmacokinetics 204. While a majority of studies does include these controls, some control formulations do not include scrambled aptamer sequences or are completely missing.

Almost all studies in this review - and in fact the vast majority of all *in vivo* imaging studies with aptamers - are in the field of oncology 39. It is therefore not surprising to see a large variability in size and location of the tumor xenografts that are used for preclinical models in these studies.

Irrespective of the obvious differences in resolution and sensitivity of the imaging modality itself, other factors that add to inter-study variabilities are the choice of the imaging agent, i.e., excitation/emission of the fluorophore or QDs, energy and half-life of SPECT isotopes, positron range of PET isotopes, Raman band shift of SERS reporters and other properties of the reporter molecule. Where present, the variability of other components such as size, shape and charge of nanocarriers further aggravate the inter-study variability. This makes a direct comparison of different probes nearly impossible, hence it is important to characterize and evaluate each probe thoroughly.

2) A majority of the literature about aptamers used for *in vivo* imaging is characterized by a lack of quantitative data. Often times only representative images are shown, without further image analysis or quantification of organ uptake. This is especially prevalent for fluorescent imaging, where more often than not the fluorescent images of whole mice are presented, and the reader is left with interpreting the results. While quantitative analyses of *in vivo* fluorescence images might be not as straight forward as for PET, it is by no means impossible and at the very least semi-quantitative analyses would allow for better comparison of fluorescently labeled aptamer probes and their respective controls.

Similarly, we find that some theranostic probes clearly focus on the therapeutic effect, which could explain why in some cases image analysis is not performed thoroughly. Nevertheless, claiming theranostic capability for a probe should naturally come with adequate analysis to support such claims. Although qualitative imaging is certainly valuable during the discovery and selection of aptamers and aptamer-based probes, more information such as tumor-to-background signal, tumor uptake levels, relative change in contrast and similar data is needed to truly evaluate novel imaging probes and compare different studies among each other.

3) Improvements of the original SELEX process have greatly increased the likelihood of success and reduced time and cost of the selection process. Especially cell-SELEX has become a powerful tool for the selection of aptamers that specifically bind cancer cells with high specificity 205. One shortcoming, however, is that in the majority of cases the molecular target of the candidate sequence is not known. A notable exception to this is sgc8/sgc8c, which has been developed by cell-SELEX against CCRF-CEM cells 42 and for which PTK7 was identified as the molecular target 206. Since then, sgc8/sgc8c have been among the most used aptamers for drug targeting, imaging and diagnostics and remain among a few promising candidates for clinical use. Regrettably, the same cannot be said for the majority of aptamers developed by cell-SELEX which are often reported without any follow-up studies. To evaluate novel aptamers for clinical use beyond recognition of cells they have been selected against, a thorough characterization of the target molecule is required. This is a time and resource intensive undertaking, and the help of the pharmaceutical industry, and industrial or academic commercialization and research centers would be very welcome in the aptamer field. A growing number of potential sequences for a variety of targets in cancer or other diseases are available, which harbors great potential for the development of commercially successful aptamer therapeutic and diagnostic products.

As a result of the differences in study parameters and lack of standardized evaluation, the imaging outcome is often difficult to interpret, and it is unclear if aptamer specificity and affinity or study design differences are to blame. In a recent systematic analysis of a panel of commonly used aptamers and cell lines, Kelly *et al*. established a number of important findings that further highlight the need for standardized assay conditions and test protocols to distinguish “real” binding sequences from non-specifically binding sequences 207. Notably, only 4 out of 11 tested aptamers demonstrated specific binding under normalized assay conditions. Even more concerningly, *in vitro* binding did not translate into reliable *in vivo* tumor labeling, which further complicates the identification of aptamers for targeted drug delivery and imaging. Improving on the just discussed key factors and adopting stringent characterization of novel sequences will help to put the role of aptamers into its well-deserved place and advance them from infancy to a stage closer to preclinical research.

In conclusion, the proven and postulated advantages of aptamers will allow for the development of powerful imaging probes, and it is only a matter of time until aptamers will find their way into the clinics. As summarized in tables 1 and 2 aptamers are now used in virtually all important imaging modalities including newer developments such as SERS imaging. With the emergence of “smart” imaging probes, fluorescent aptamers have also entered an exciting new avenue that could potentially lead to interesting developments beyond early preclinical evaluation studies of fluorescent molecular imaging probes.

## Figures and Tables

**Table 1:** Molecular Imaging with aptamers. S. = subcutaneous, O. = orthotopic, * = novel aptamer, ᶧ = from company, ^1^ = DLS, ^2^ = TEM, ^a^ = unconjugated control, ^b^ = scrambled oligonucleotide conjugated control

Target	Name	Chemistry	Multimodal Imaging	Imaging	Nanocarrier	Size	Reporter molecule	Preclinical Model	Comments	Reference
Nucleolin	AS1411	DNA	No	MRI	Polyrotaxane	Not reported	Gd-DTPA	S. MCF-7	Quantitative *in vivo* imaging^a^.	77
Endoglin (CD105)	Apt1*	DNA	Yes	MRI/Fluorescence	PAMAM dendrimers	6.3 nm^1^ (non-targeted)/7.5 nm^1^ (targeted)	Gd-DTPA/IR783	O. SMMC-7721-GFP	Quantitative *in vivo* imaging^a^, fluorescence only *ex vivo*	55
Nucleolin	AS1411	DNA	No	MRI	BSA coated with PDDA and mesoporous silica	345.6 nm^1^ (non-targeted)	Gd_2_O_3_/Fe_3_O_4_ (Dual T_1_ and T_2_ contrast agent)	Tested only in healthy mice (non-aptamer targeted)	Aptamer-targeted probe only evaluated *in vitro*.	80
Nucleolin	AS1411	DNA	No	MRI	MnO nanoparticles	15.1 nm^1^/1.9 nm^2^ (non-targeted)	Mn^2+^	S. 768-O	No direct comparison between targeted and non-targeted nanoparticles	81
Nucleolin	AS1411	DNA	Yes	MRI/Fluorescence	Mn-doped MoS_2_ QDs	6.1 nm^2^ (non-targeted)	Mn^2+^/quantum dots (λ_ex_ 450 nm/λ_em_ 790 nm)	S. 768-O	Quantitative *in vivo* imaging^a^. Fluorescence not evaluated *in vivo.*	82
Nucleolin	AS1411	DNA	Yes	MRI/Fluorescence	MoS_2_ QDs supported on MnO_2_ nanosheets	Only size of quantum dots reported (1.4 nm^2^)	Mn^2+^/quantum dots (λ_ex_ 300 nm/λ_em_ 398 nm)	S. 768-O	Quantitative *in vivo* imaging^a^. Fluorescence not evaluated *in vivo.*	83
HER2	APT_HER2_ᶧ	DNA (NapdU-modified)	No	MRI	Magnetic nanocrystals	28.8 nm^1^/10.5 nm^2^ (non-targeted) 34.1 nm^1^ (targeted)	Fe_3_O_4_	S. NIH3T6.7 (syngeneic)	Quantitative in vivo imaging^a^.	84
GPC3	AP613-1*	DNA	No	MRI	USPIO nanoparticles	38.0 nm^1^/10 nm^2^ (non-targeted)/45.2 (targeted)	Fe_3_O_4_	S. Huh-7	Quantitative *in vivo* imaging^a^.	56
EpCAM	Eppc6	DNA	No	MRI	GoldMag NPs	Not reported	GoldMag NPs	S. PC-3	Quantitative *in vivo* imaging^b^.	86
MUC1	S1.3/S2.2	DNA	No	SPECT	PEGylated PAMAM dendrimers	63.2 nm^1^	^67^Ga	S. MCF-7 in rats	No controls, *in vivo* imaging qualitative only.	89
VEGF-A	RNV66	DNA (locked nucleic acid-modified	No	PET	Hyperbranched polyethylene glycol	7.2 nm^1^ (targeted)	^89^Zr	S. MDA-MB-231	Quantitative *in vivo* imaging^a^.	90
EGFR	ME07	RNA (2'F-modified)	No	PET	-	^-^	^18^F	S. A431, U87MG and HCT-116	Excellent experimental design for *in vivo* imaging, quantitative imaging. No scrambled ssRNA control	92
HER2	SH-1194-35ᶧ	DNA (NapdU-modified)	No	PET	-	^-^	^18^F	S. BT474 (HER2^+^) and MB-MDA-231 (HER2^-^)	Quantitative *in vivo* imaging, no scrambled ssDNA control	51
Fibronectin	AS-14	DNA	No	PET	-	-	^11^C	Metastatic Ehrlich ascites carcinoma model	Good controls, semiquantitative imaging	93
EpCAM	#1-F	DNA	No	PET	PEG2000 linker	Not reported	^64^Cu	S. MDA-MB-231 (EpCAM^+^) and U937 (EpCAM^-^)	Quantitative imaging with good controls. No scrambled ssDNA control	95
PTK7	sgc8	DNA	No	PET	Gold nanoclusters	94 nm^2^	^68^Ga	S. HCT-116	Qualitative *in vivo* imaging, free ^67^Ga-Sgc8 control	96
Nucleolin/MUC1	AS1411/S2.2	DNA	No	SERS	Gold NPs	69 nm^1^/70 nm^2^ (targeted)	1,4-diphenylbuta-1,3-diyne (Raman shift 2205 cm^-1^)	S. MCF-7	Qualitative *in vivo* imaging^b^.	107
Nucleolin/*(RGD-peptide/CD44 antibody)*	AS1411	DNA	No	SERS	Gold NPs	132.3 nm^1^ (RGD), 135.3 nm^1^ (aptamer), 138.6 nm^1^ (CD44)	1-azido-4-ethynylbenzene (Raman shift 2120 cm^-1^)	S. MDA-MB-231 and MCF-7 cells	Multiplexed probe to distinguish CD44 expression profile* in vivo* using also RGD-peptide and CD44 antibody targeted nanoparticles. Non-targeted nanoparticles used as control	108
Nucleolin	AS1411	DNA	No	Spontaneous Raman emission	Poly(methacrylate) NPs	100 nm^1^ (targeted)	1,4-diphenylbuta-1,3-diyne (Raman shift 2205 cm^-1^)	S. MCF-7	Qualitative *in vivo* imaging. RGD-peptide and non-targeted NPs as control.	110
Carbonic anhydrase IX	CAIX aptamer*	DNA	No	US	Lipid nanobubbles	484 nm^1^ (non-targeted)/478 nm^1^ (targeted)	Perfluoropropane	S. 786-O and HeLa (CAIX positive) and BxPC-3 (CAIX negative)	Quantitative *in vivo* imaging^b^.	57
Nucleolin	AS1411	DNA	No	US	Lipid nanobubbles	533.5 nm^1^ (targeted)/459.3 nm^1^ (non-targeted)	Perfluoropropane	S. MDA-MB-231 and MDA-MB-468	Quantitative *in vivo* imaging^a^.	117
PSMA	A10-3.2	RNA	No	US	Multi-walled carbon nanotubes	400 nm length, 15 nm diameter^1^ (non-targeted)/30 nm diameter (targeted)	Carbon nanotubes	S. PC-3	Qualitative *in vivo* imaging^a^.	118
GPC3	A613-1*/AP613-1*	DNA/DNA (phosphorothioate-modified)	No	Fluorescence	-	-	Alexa Fluor 750	S. bilateral Huh-7 (GPC3-positive) and A549 (GPC3-negative)	Good *in vivo* model to confirm specificity^b^. Quantitative *in vivo* imaging.	44, 45
lung-metastatic osteosarcoma	LP-16*	DNA	No	Fluorescence	-	-	Cy5	S. 143B	Qualitative *in vivo* imaging^b^.	50
Multidrug resistant HCC	PS-ZL-7c*	DNA (phosphorothioate-modified	No	Fluorescence	-	-	Cy5	S. bilateral HepG2/MDR and HepG2	Good *in vivo* model to confirm specificity^b^. Qualitative *in vivo* imaging.	49
HER-2 enriched breast cancer	Sk6Ea*	DNA	No	Fluorescence	-	-	Cy5	S. SK-BR-3 (target cell line), MCF-7 and MDA-MB-231	Qualitative *in vivo* imaging^b^. Histochemistry of patient derived cancer tissue	46
Luminal A subtype breast cancer	MF3/MF3Ec*	DNA	No	Fluorescence	-	-	Cy5	S. MCF-7 (target cells), SK-BR-3 and MDA-MB-231	Qualitative *in vivo* imaging^b^. Histochemistry of xenograft cancer tissue	47, 48
Papillary thyroid carcinoma	TC-6*	DNA	No	Fluorescence	-	-	Cy5	S. TPC-1	Qualitative *in vivo* imaging^b^. Histochemistry of patient derived cancer tissue	52
Vemurafenib-resistant melanoma (CD63)	LL4A*	DNA	No	Fluorescence	-	-	Cy5	S. bilateral Mel28 and MEL28-PLX (target cells)	Good *in vivo* model to confirm specificity^b^. Qualitative *in vivo* imaging.	51
Alkaline phosphatase heterodimers	BG2*	DNA (phosphorothioate-modified)	No	Fluorescence	-	-	Alexa Fluor 647	S. LoVo (AP-positive) and PC-3 (AP-negative)	Qualitative in vivo imaging^b^.	53
Broad cancer specificity	E3	RNA (2'-F-modified pyrimidines and 2' OH purines)	No	Fluorescence	-	-	Alexa Fluor 750	S. CRC119x	Qualitative in vivo imaging^b^.	54
Ovarian cancer cells	R13	DNA	No	Fluorescence	-	-	Cy5	S. A2780	Qualitative in vivo imaging^b^.	55
PTK7/SMMC-7721 cells	sgc8c/Zy11	DNA	No/pH-activatable	Fluorescence	-	-	Cy5	S. CCRF-CEM (target) and SMMC-7721 (control)	Intratumoral injection, *in vivo* imaging qualitative only, no untargeted controls	136
A549 cells	S6	DNA	No/pH-activatable	Fluorescence	-	-	Cy5	S. A549 (target) and SMMC-7721 (control)	Qualitative *in vivo* imaging. Good controls	138
Transferrin receptor 1 (CD71)	XQ-2d	DNA	No	Fluorescence	PEG_5000_ *via* hypoxia-cleavable azobenzene linker	Not reported	Cy5	S. DU145	Qualitative *in vivo* imaging. Good controls	140
PTK7	sgc8	DNA	No	Fluorescence	Human serum albumin	Not reported	Cy5	S. HCT-116	Qualitative *in vivo* imaging. Good controls	132
CCK-B receptor	AP1153	DNA	No	Fluorescence	Calcium phosphosilicate NPs	76.7 nm^2^ (non-targeted), 79.4 nm^2^ (targeted)	ICG	S. PANC-1 *(in vivo* imaging) and PC-3	Qualitative in vivo imaging^a^.	135
EGFR	CL4	RNA (2'F-uracil-modified)	Yes	MRI/Fluorescence	DNA nanotriangle	33.4 nm^1^ (targeted)	Gd-DTPA/DyLight 800	S. MDA-MB-231	Quantitative *in vivo* fluorescence imaging^a^, MRI qualitative only with Gd-DOTA control	129
ATP	Unknown, from 141, 142	DNA	No	Fluorescence	NaGdF4:70%Yb,1%Tm@NaGdF4 NPs	40 nm^2^	Cy5	S. HeLa	Intratumoral injection, quantitative *in vivo* imaging.	143
ATP	Unknown, from 141, 142	DNA	No	Fluorescence	NaGdF4:70%Yb,1%Tm@NaGdF4 NPs	45 nm^2^	Cy5	S. HeLa	Excellent controls, quantitative *in vivo* imaging.	144
ATP	Unknown, from 141, 142	DNA	No	Fluorescence	Honeycomb MnO_2_ NPs	144 nm^2^ (targeted)	Cy5	S. 4T1	Intratumoral injection, quantitative *in vivo* imaging, no stringent controls	145
ATP	Unknown, from 141, 142	DNA	No	Fluorescence	Titanium carbide nanosheets	200-300 nm^2^	ROX	S. 4T1 and MCF-7	Intratumoral injection, quantitative *in vivo* imaging, no stringent controls	146

**Table 2:** Theranostic Probes with aptamers. S. = subcutaneous, O. = orthotopic, * = novel aptamer, ᶧ = from company, ^1^ = DLS, ^2^ = TEM, ^a^ = unconjugated control, ^b^ = scrambled oligonucleotide conjugated control

Target	Name	Chemistry	Imaging	Therapy	Nanocarrier	Size	Reporter Molecule	Preclinical Model	Comments	Reference
Nucleolin	AS1411	DNA	MRI/Fluorescence	Sonodynamic Therapy - Nearly complete growth inhibition, non-targeted probe only slightly worse	MnO_2_ coated Liposomes encapsulating HMME and ACF	187.7 nm^1^ (non-targeted)/ 185.4 nm^1^ (targeted)	Mn^2+^/IR780	S. SKOV-3	Qualitative in vivo imaging^a^.	180
Fibronectin/Hsc70	AS-14/AS-42	DNA	MRI	MDT - complete tumor necrosis for aptamer-targeted formulation, reduced effect for non-targeted formulation	Ferroarabinogalactan nanoparticles	1.96 µM^2^	Superparamagnetic iron oxide	S. and intracranial Ehrlich ascites carcinoma	Qualitative *in vivo* imaging, only Omniscan^TM^ control.	175
Nucleolin	AS1411	DNA	MRI/Fluorescence	DOX, HSP70/HSP90 siRNA/MDT therapy - Excellent tumor growth inhibition, no comparison to non-targeted probe.	Zinc-doped iron oxide octahedra with PAMAM and PEG modification	41.1 nm^1^ (without aptamer functionalization)	Superparamagnetic iron oxide/Cy5.5	S. 4T1	Quantitative *in vivo* imaging^b^.	178
MUC1	S1.3/S2.2	DNA	MRI	DOX - moderate tumor growth inhibition for targeted probe. No difference between non-targeted probe and free DOX	Pyoverdine coated SPIONs	96.5 nm^1^ (non-targeted), 119.8 nm^1^ (targeted), 127.6 nm^1^ (aptamer conjugated with DOX)	Fe^2+^/Fe^3+^	S. C26	Qualitative *in vivo* imaging^a^.	172
Nucleolin	AS1411	DNA	MRI	Deferasirox - moderate tumor growth inhibition, contradictory result of Kaplan-Meier curve.	Deferasirox coated SPIONs	20-50 nm^2^	Fe^2+^/Fe^3+^	S. C26	Qualitative *in vivo* imaging^a^.	165
EpCAM	EP1	DNA	MRI/Fluorescence	DOX - Strongest tumor inhibition for targeted formulation but no statistical analysis.	Mesoporous silica coated Gd-Zn-Cu-In-S/ZnS quantum dots	100 nm^2^ (non-targeted)	Gd/quantum dots (λ_ex_ 450 nm/λ_em_ 790 nm)	S. 4T1	Qualitative *in vivo* MRI^a^, Fluorescence only evaluated *ex vivo.*	162
Nucleolin	AS1411	DNA	MRI/Fluorescence	DOX - Strongest tumor inhibition for targeted formulation but no statistical analysis.	Quantum dots from 162 loaded in PEG-PCL polymersomes	130.6 nm^1^ (non-targeted)/136.3 nm^1^ (targeted)	Gd/ quantum dots (λ_ex_ 450 nm/λ_em_ 790 nm)	S. 4T1	Qualitative *in vivo* MRI^a^, Fluorescence only evaluated *ex vivo.*	163
PSMA	A10-3.2	RNA	SPECT	MDM2 siRNA - dose dependent tumor growth inhibition	-	-	^99m^Tc	S. 22Rv1 (PSMA^+^) and PC-3 (PSMA^-^)	Qualitative *in vivo* imaging, free [^99m^Tc]TcO_4_^-^ control.	168
Nucleolin/MUC1	AS1411/S2.2	DNA	SERS	PTT - complete tumor growth inhibition for both targeted probes, no difference between untargeted control and saline.	Gold nanorods	39.5 nm^1^ (non-targeted)/44.6 nm^1^ (targeted)	1,4-diphenylbuta-1,3-diyne (Raman shift 2205 cm^-1^)	S. MCF-7	Qualitative *in vivo* imaging^b^.	109
Nucleolin	AS1411	DNA	CT	Sonodynamic therapy - Strong tumor growth inhibition with targeted probe, reduced efficacy without targeting	Au-TiO_2_ nanosheets, triphenylphosphine modified for mitochondria targeting	40 nm^2^ (TiO_2_ nanosheets only)	Gold nanoparticles	S. MCF-7	Focus on therapy, qualitative *in vivo* imaging without controls.	122
MUC1	S2.2	DNA	CT	Curcumin - Strong tumor growth inhibition with targeted probe, reduced efficacy without targeting	Poly-(amidoamine) dendrimers	4.3 nm^1^ (non-targeted)/5.2 nm^1^ (targeted)	Gold nanoparticles	S. C26	*In vitro* characterization non-conclusive, qualitative *in vivo* imaging^a^.	123
Renal cell carcinoma	SW-4b*	DNA	Fluorescence	Inhibits cell proliferation of 786-O cells with IC_50_ of 4.7 µM - evaluated only *in vitro.*	-	-	Cy5	S. 768-O	Qualitative *in vivo* imaging^b^.	58
c-met	SL1	DNA	Fluorescence	Inhibits multiple myeloma cell proliferation and migration, inhibits c-met signaling, synergistic in combination treatment with Bortezomib - *in vitro* only	-	-	Cy5	S. ARP-1	Qualitative *in vivo* imaging^b^.	155
NSCLC	RA16*	RNA (synthetic and *in vitro* transcribed from DNA template)	Fluorescence	Inhibits NSCLC cell growth *in vitro* and *in vivo*. Synergistic effect with intercalated Epirubicin	-	-	Cy5.5	S. NCL-H460 (used for imaging and *in vivo* tumor growth inhibition)	*In vivo* SELEX, qualitative *in vivo* imaging^b^.	9, 59
CD133	AP-1-M*	DNA	Fluorescence	Doxorubicin - No difference between free DOX and DOX loaded AP-1-M, but reduced cell toxicity	-	-	Cy5.5	S. FRO	Qualitative *in vivo* imaging^b^.	60
PDGFRβ	Gin4.T	RNA (2'-F Pyrimidine-modified)	Fluorescence	Inhibits growth and invasion of MDA-MB-231 and BT-549 cells *in vitro*, Reduces number of metastatic foci and tumor growth of MDA-MB-231 tumors *in vivo*	-	-	VivoTag-S 680	S. MDA-MB-231 and BT474 for *in vivo* tumor imaging studies. Lung metastatic model of intravenously injected MDA-MB-31-GFP cells.	Excellent controls and study design for *in vivo* imaging as well as therapy, quantitative in vivo imaging^b^.	158
EGFR	CL4	RNA (2'F-uracil-modified)	Fluorescence	Cisplatin - strong tumor growth inhibition compared to free cisplatin or non-targeted NPs	PLGA-PEG polymeric nanoparticles	107.9 nm^1^ (aptamer targeted), 104.2 nm^1^ (control sequence), 90.5 nm^1^ (non-targeted)	Cy7	S. MDA-MB-231	Quantitative *in vivo* imaging^a,b^.	197
Nucleolin	AS1411	DNA	Fluorescence	Triptolide - strong tumor growth suppression of aptamer triptolide conjugate, no effect of free triptolide	-	-	Cy5	S. MDA-MB-231	Qualitative *in vivo* imaging^b^.	166
Nucleolin	AS1411	DNA	Fluorescence	PROTAC - strong tumor growth inhibition for APC, moderate improvement compared to free PROTAC	-	-	Cy3	S. MCF-7	Qualitative *in vivo* imaging^b^.	169
PTK7	sgc8c	DNA	Fluorescence, target recognition activatable	DOX - strong growth inhibition from DOX alone, slightly better with targeted nanoformulation.	DNA-nanotriangle	10.2 nm^1^ (non-targeted)/33.7 nm^1^ (targeted)	Cy5	S. CCRF-CEM and Ramos	Qualitative *in vivo* imaging^b^.	185
Nucleolin	AS1411	DNA	Fluorescence	Singlet oxygen (^1^O_2_) from hemin and Pyrochlorophyll A - strong tumor growth inhibition and reduction of hypoxia *in vivo*.	Hemin intercalated in sequence	10 nm^1^	Pyrochlorophyll A	S. MCF-7	Quantitative in vivo imaging, no stringent controls for imaging.	186
Nucleolin	AS1411	DNA	Fluorescence, Photothermal imaging	DOX, ICG (singlet oxygen (^1^O_2_) production) - strong tumor growth inhibition, missing controls	BSA	116 nm^1^ (non-targeted), targeted probe slightly larger but exact size not reported	ICG	S. MCF-7	Qualitative in vivo imaging, no stringent controls.	164
EGFR	Unknownᶧ	Unknown	Fluorescence	Paclitaxel - Comparable tumor growth inhibition of aptamo-QDs and immuno-QDs, reduced effect for non-targeted NPs.	LNPs	40.5 nm^1^ (non-targeted), 41.5 nm^1^ (aptamo-QD) and 42.8 nm^1^ (immuno-QD	Quantum dots (λ_ex_ 540 nm/λ_em_ 620 nm)	S. LS174T	Qualitative *in vivo* imaging using aptamer and antibody targeted formulation, excellent *ex vivo* quantification.	173
EGFR	Unknownᶧ	Unknown	Fluorescence	Bcl-2 & PKC-ι siRNA - Comparable tumor growth inhibition of aptamo-QDs and immuno-QDs, reduced effect for non-targeted NPs	LNPs	160.4 nm^1^ (non-targeted), 171.7 nm^1^ (aptamo-QD) and 175.5 nm^1^ (immuno-QD	Quantum dots (λ_ex_ 540 nm/λ_em_ 620 nm)	S. MDA-MB-231	Qualitative *in vivo* imaging using aptamer and antibody targeted formulation, excellent *ex vivo* quantification.	174
Nucleolin	AS1411	DNA	Fluorescence/MRI	PTT - excellent tumor growth inhibition, no difference between targeted and non-targeted nanoprobe	Gold nanobipyramid	75 x 27 nm^2^	Gd_2_O_3_ (MRI) and gold nanoclusters (fluorescence)	S. MDA-MB-231	Quantitative *in vivo* MRI^a^, fluorescence not evaluated in vivo.	179
Nucleolin	AS1411	DNA	Photoacoustic imaging/US	PTT - complete tumor remission for targeted NPs, slightly reduced effect for non-targeted NPs	PLGA coated iron(II) phthalocyanine (FePC) and liquid perfluoropentane	185.1 nm^1^ (non-targeted), 201.9 nm^1^ (targeted)	FePC (photoacoustic imaging/perfluoropentane (US)	S. MCF-7	Quantitative *in vivo* imaging^a^.	181
Nucleolin	AS1411	DNA	Fluorescence	DOX - Statistically significant best growth inhibition with nanoprobe, strong effect also with free DOX and aptamer-DOX conjugate	Hyaluronic acid encapsulation	108 nm^1^/112 x 16 nm^2^	Doxorubicin autofluorescence	S. 4T1	Qualitative *in vivo* imaging without stringent controls.	187
LH86 hepatocellular carcinoma cells	TLS11a	DNA	Fluorescence	β-lapachone and tirapazamine - strong tumor growth inhibition but small effect of targeting	PEG-PO-PCL-PO-PEG polymeric nanoparticle	220 nm^1^/200 nm^2^ (targeted)	ICG	S. Hep2G	Quantitative *in vivo* imaging^b^.	188
PTK7	sgc8	DNA	Fluorescence	PDT - excellent tumor growth inhibition with nanoprobe + NIR, no effect for probe without NIR.	Multi shell upconversion luminescence nanoparticles	54.1 nm^1^	Cy3/Cy5	dimethylbenzanthracene-induced breast cancer	Good controls, qualitative *in vivo* imaging.	183
Nucleolin	AS1411	DNA	Fluorescence	Radiation therapy - significant increase of median survival time for mice treated with targeted NPs	Silver nanoparticles	37.8 nm^1^ (targeted, 33.3 nm^1^ (non-targeted)	Cy5	Intracranial C6 glioma xenograft	Quantitative in vivo imaging^a^,	182
Nucleolin	AS1411	DNA	Fluorescence/MRI	PDT/chemodynamic therapy/PTT- strong tumor growth inhibition, small difference between targeted and non-targeted group	MnO_2_ encapsulated gold nanoclusters	136.3 nm^1^	ICG (fluorescence)/Mn^2+^ (MRI)	S. MCF-7	Qualitative *in vivo* imaging without stringent controls.	189
ATP	Unknown, from 141, 142	DNA	Fluorescence	PDT - excellent tumor growth inhibition for formulation, regardless of presence of heme	Black phosphorous nanosheets	115 nm with 3.8 nm thickness^2^	Cy5	S. HeLa	Qualitative *in vivo* imaging, no controls.	193
ATP	Unknown, from 141, 142	DNA	Fluorescence	PDT - strong tumor growth inhibition for formulation + NIR, no effect for treatment with Let7a inhibitor and formulation or control groups	CdTe/ZnS quantum dots	67.7 nm^1^ (targeted)	Quantum dots 555 and 627 nm dal emission peak	S. MCF-7	Intratumoral injection, quantitative *in vivo* imaging.	194

## Abbreviations

ADC: aptamer drug conjugate
APC: aptamer protac conjugate
ARMD: age-related macular degeneration
ATP: adenosine triphosphate
ATU: 3,9-bis(3-aminopropyl)-2,4,8,10-tetraoxaspiro [5.5]undecane
BSA: bovine serum albumin
CAIX: carbonic anhydrase IX
CCK-B: cholecystochinin-B
CT: computed tomography
DNA: deoxyribonucleic acid
DOTA: dodecane tetraacetic acid
DOX: doxorubicin
DTPA: diethylenetriamine pentaacetate
EGFR: endothelial growth factor receptor
EPR: enhanced permeability and retention
FePC: iron(II) phthalocyanine
GPC3: glypican-3
GSH: glutathione
HCC: hepatocellulary carcinoma
ICG: indocyanine green
MDT: magnetodynamic therapy
MRI: magnetic resonance imaging
NP: nanoparticle
NSCLC: non-small-cell lung carcinoma
PAMAM: poly(amidoamine)
PEG: polyethylene glycol
PDT: photodynamic therapy
PET: positron emission tomography
p.i.: post injection
PTT: photothermal therapy
PROTAC: proteolysis targeting chimeras
PTC: papillary thyroid carcinoma
PTK7: tyrosine-protein kinase-like 7
QD: quantum dot
QDM: quantum dot micelles
RNA: ribonucleic acid
ROS: reactive oxygen species
SELEX: systematic evolution of ligands by exponential enrichment
SERS: surface enhanced Raman scattering
SILAC: stable isotope labeling with amino acids in cell culture
SPECT: single-photon emission computed tomography
TME: tumor microenvironment
TNBC: triple-negative breast cancer
US: ultrasound imaging
VEGF: vascular endothelial growth factor
